# New-onset and relapsed liver diseases following COVID-19 vaccination: a systematic review

**DOI:** 10.1186/s12876-022-02507-3

**Published:** 2022-10-13

**Authors:** Saad Alhumaid, Abbas Al Mutair, Ali A. Rabaan, Fatemah M. ALShakhs, Om Prakash Choudhary, Shin Jie Yong, Firzan Nainu, Amjad Khan, Javed Muhammad, Fadil Alhelal, Mohammed Hussain Al Khamees, Hussain Ahmed Alsouaib, Ahmed Salman Al Majhad, Hassan Redha AL-Tarfi, Ali Hussain ALyasin, Yaqoub Yousef Alatiyyah, Ali Ahmed Alsultan, Mohammed Essa Alessa, Mustafa Essa Alessa, Mohammed Ahmed Alissa, Emad Hassan Alsayegh, Hassan N. Alshakhs, Haidar Abdullah Al Samaeel, Rugayah Ahmed AlShayeb, Dalal Ahmed Alnami, Hussain Ali Alhassan, Abdulaziz Abdullah Alabdullah, Ayat Hussain Alhmed, Faisal Hussain AlDera, Khalid Hajissa, Jaffar A. Al-Tawfiq, Awad Al-Omari

**Affiliations:** 1 Administration of Pharmaceutical Care, Al-Ahsa Health Cluster, Ministry of Health, Rashdiah Street, P. O. Box 12944, Al-Ahsa, 31982 Saudi Arabia; 2 Research Center, Almoosa Specialist Hospital, Al-Ahsa, Saudi Arabia; 3 College of Nursing, Princess Norah Bint Abdul Rahman University, Riyadh, Saudi Arabia; 4 School of Nursing, University of Wollongong, Wollongong, Australia; 5 Molecular Diagnostic Laboratory, Johns Hopkins Aramco Healthcare, Dhahran, Saudi Arabia; 6 College of Medicine, Alfaisal University, Riyadh, 11533 Saudi Arabia; 7 Department of Public Health and Nutrition, The University of Haripur, Haripur, Pakistan; 8 Respiratory Therapy Department, Prince Saud Bin Jalawi Hospital, Ministry of Health, Al-Ahsa, Saudi Arabia; 9 Department of Veterinary Anatomy and Histology, College of Veterinary Sciences and Animal Husbandry, Central Agricultural University (I), Selesih, Aizawl, Mizoram 796015 India; 10 Department of Biological Sciences, School of Medical and Life Sciences, Sunway University, Subang Jaya, Malaysia; 11 Department of Pharmacy, Faculty of Pharmacy, Hasanuddin University, Makassar, 90245 Indonesia; 12 Department of Microbiology, The University of Haripur, Haripur, 22620 Khyber Pakhtunkhwa Pakistan; 13 Optometry Department, Dhahran Eye Specialist Hospital, Ministry of Health, Dhahran, Saudi Arabia; 14 Molecular Pathology Laboratory, King Fahad Hofuf Hospital, Ministry of Health, Al-Ahsa, Saudi Arabia; 15 Medical Store Department, Maternity and Children Hospital, Ministry of Health, Al-Ahsa, Saudi Arabia; 16 Department of Pharmacy, Hereditary Blood Diseases Centre, Al-Ahsa, Saudi Arabia; 17 Medical Supply Store, Aloyoon General Hospital, Ministry of Health, Al-Ahsa, Saudi Arabia; 18 Inventory Control Unit, Aloyoon General Hospital, Ministry of Health, Al-Ahsa, Saudi Arabia; 19 Pharmacy Department, Aloyoon General Hospital, Ministry of Health, Al-Ahsa, Saudi Arabia; 20 Pharmacy Department, Prince Saud Bin Jalawi Hospital, Ministry of Health, Al-Ahsa, Saudi Arabia; 21 Pharmacy Department, King Fahad Hofuf Hospital, Ministry of Health, Al-Ahsa, Saudi Arabia; 22 Pharmacy Department, Maternity and Children Hospital, Ministry of Health, Al-Ahsa, Saudi Arabia; 23 Administration of Nursing Care, Maternity and Children Hospital, Ministry of Health, Al-Ahsa, Saudi Arabia; 24 General Surgery Department, King Fahad Hofuf Hospital, Ministry of Health, Al-Ahsa, Saudi Arabia; 25 Department of Medical Microbiology and Parasitology, School of Medical Sciences, Universiti Sains Malaysia, 16150 Kubang Kerian, Kelantan Malaysia; 26 Infectious Disease Unit, Specialty Internal Medicine, Johns Hopkins Aramco Healthcare, Dhahran, Saudi Arabia; 27 Infectious Disease Division, Department of Medicine, Indiana University School of Medicine, Indianapolis, IN USA; 28 Infectious Disease Division, Department of Medicine, Johns Hopkins University School of Medicine, Baltimore, MD USA; 29 College of Medicine, Alfaisal University, Riyadh, Saudi Arabia; 30 Research Center, Dr. Sulaiman Al Habib Medical Group, Riyadh, Saudi Arabia

**Keywords:** SARS-CoV-2, COVID-19, Disease, Hepatic, Liver, Pathology, Safety, Side effect, Systematic review, Vaccine, Vaccination

## Abstract

**Background:**

Liver diseases post-COVID-19 vaccination is extremely rare but can occur. A growing body of evidence has indicated that portal vein thrombosis, autoimmune hepatitis, raised liver enzymes and liver injuries, etc., may be potential consequence of COVID-19 vaccines.

**Objectives:**

To describe the results of a systematic review for new-onset and relapsed liver disease following COVID-19 vaccination.

**Methods:**

For this systematic review, we searched Proquest, Medline, Embase, PubMed, CINAHL, Wiley online library, Scopus and Nature through the Preferred Reporting Items for Systematic Reviews and Meta Analyses PRISMA guideline for studies on the incidence of new onset or relapsed liver diseases post-COVID-19 vaccination, published from December 1, 2020 to July 31, 2022, with English language restriction.

**Results:**

Two hundred seventy-five cases from one hundred and eighteen articles were included in the qualitative synthesis of this systematic review. Autoimmune hepatitis (138 cases) was the most frequent pathology observed post-COVID-19 vaccination, followed by portal vein thrombosis (52 cases), raised liver enzymes (26 cases) and liver injury (21 cases). Other cases include splanchnic vein thrombosis, acute cellular rejection of the liver, jaundice, hepatomegaly, acute hepatic failure and hepatic porphyria. Mortality was reported in any of the included cases for acute hepatic failure (n = 4, 50%), portal vein thrombosis (n = 25, 48.1%), splanchnic vein thrombosis (n = 6, 42.8%), jaundice (n = 1, 12.5%), raised liver enzymes (n = 2, 7.7%), and autoimmune hepatitis (n = 3, 2.2%). Most patients were easily treated without any serious complications, recovered and did not require long-term hepatic therapy.

**Conclusion:**

Reported evidence of liver diseases post-COIVD-19 vaccination should not discourage vaccination against this worldwide pandemic. The number of reported cases is relatively very small in relation to the hundreds of millions of vaccinations that have occurred and the protective benefits offered by COVID-19 vaccination far outweigh the risks.

## Background

Vaccinations against coronavirus disease 2019 (COVID-19) is a crucial step in ending the current worldwide pandemic. Vaccines such as Pfizer-BioNTech, Oxford Uni-AstraZeneca, Moderna, Johnson & Johnson, Sinovac-CoronaVac, Covishield, and Sinopharm have been developed rapidly, determined as safe, approved under emergency use authorization since early 2020 and had been used widely. As of 1 May 2022, there have been more than 5 billion of severe acute respiratory syndrome coronavirus 2 (SARS-CoV-2) vaccine doses administered globally [1]. Therefore, new safety, adverse effects, or toxicity concerns related to the COVID-19 vaccination have emerged. Adverse reactions to COVID-19 vaccines are commonly reported, but most are not hepatically mediated. Localized pain, fatigue, headache and muscle ache are the most prevalent adverse effects following COVID-19 vaccination [2]. Liver toxicity is rare with all vaccines used to prevent COVID-19, but can occur. A growing body of evidence has indicated that portal vein thrombosis [3–5], autoimmune hepatitis [6–8], raised liver enzymes [9–11] and liver injuries [12, 13], etc., may be potential consequence of COVID-19 vaccines. COVID-19 vaccines are usually administered in 2- or 3-dose series over a short time only [14, 15], and the symptoms and signs of the COVID-19 infection overshadow the mild and transient liver adverse effects that arises with some of the vaccines used to prevent COVID-19. Furthermore, instances of acute hepatitis [16], raised liver enzymes [17, 18] and liver injury [19] have been reported in patients with moderate and severe COVID-19 in which vaccines did not appear to play a role. Whether the association between SARS-CoV-2 vaccines and those liver diseases is coincidental or causal remains to be elucidated.

In light of newer case reports and case-series studies that were published to describe the incidence of hepatotoxicity in patients who received the COVID-19 vaccines, we provide a systematic review of the current literature to delineate the range of liver diseases that were elicited following COVID-19 vaccination. We expect our review to provide clinicians with a thorough understanding of these rare adverse events.

## Methods

### Design

We followed the Preferred Reporting Items for Systematic Reviews and Meta-Analyses guidelines PRISMA in conducting this systematic review [20]. The following electronic databases were searched: PROQUEST, MEDLINE, EMBASE, PUBMED, CINAHL, WILEY ONLINE LIBRARY, SCOPUS and NATURE with Full Text. We used the following keywords: (“*COVID-19*” OR “*SARS-CoV-2*” OR “*Severe acute Respiratory Syndrome Coronavirus 2*” OR “*Coronavirus Disease 2019*” OR “*2019 novel coronavirus*”) AND *vaccine* OR *vaccination* AND (“*liver histopathology*” OR “*liver disease*” OR “*hepatic disease*” OR “*liver toxicity*” OR “*hepatotoxicity*”). The search was limited to papers published in English between 1 December 2020 and 31 July 2022. Based on the title and abstract of each selected article, we selected those discussing and reporting occurrence of new-onset or relapsed liver disease following SARS-CoV-2 vaccination.

### Inclusion–exclusion criteria

The inclusion criteria are as follows: (1) published case reports, case series and cohort studies that focused on new-onset or relapsed liver diseases following SARS-CoV-2 vaccination that included adults as population of interest; (2) studies of experimental or observational design reporting the incidence of new-onset or relapsed liver diseases in patients post-SARS-CoV-2 vaccination; and (3) the language was restricted to English. The exclusion criteria are as follows: (1) studies that did not report data on new-onset or relapsed liver diseases due to SARS-CoV-2 vaccination; (2) studies that did not report details on identified new-onset or relapsed liver disease cases following COVID-19 vaccination; (3) studies that reported new-onset or relapsed liver disease in patients with no history of COVID-19 vaccination; and (4) duplicate publications.

### Data extraction

Six authors (Saad Alhumaid, Abbas Al Mutair, Ali Rabaan, Fatemah M. ALShakhs, Shin Jie Yong, and Hussain Ahmed Alsouaib) critically reviewed all of the studies retrieved and selected those judged to be the most relevant. Data were carefully extracted from the relevant research studies independently. Articles were categorized as case report or case-series studies. The following data were extracted from selected studies: authors; publication year; study location; study design and setting; age; proportion of male patients; patient ethnicity; time to hospital presentation with liver pathology from day of vaccination, medical comorbidities; vaccine brand and dose (if 1st dose, 2nd dose or 3rd dose); if liver pathology is new-onset or relapsed; patient clinical presentation; abnormal laboratory indicators; biopsy examination and radiological imaging findings; treatment given; assessment of study risk of bias; and treatment outcome (survived or died); which are noted in Table 1.

### Quality assessment

The quality assessment of the studies was undertaken mainly based on the modified Newcastle–Ottawa Scale (NOS) to assess the quality of the selected studies [21]. Items related to the comparability and adjustment were removed from the NOS and items which focus on selection and representativeness of cases, and ascertainment of outcome and exposure are kept [22]. Modified NOS consists of five items each requires yes and no response to indicate whether bias was likely, and these items were applied to single-arm studies [22]. Quality of the study was considered good if all five criteria were met, moderate when four were met, and poor when three or less were met. Quality assessment was performed by six authors (Mohammed Hussain Al Khamees, Yaqoub Yousef Alatiyyah, Ali Ahmed Alsultan, Hassan N. Alshakhs, Haidar Abdullah Al Samaeel, and Rugayah Ahmed AlShayeb) independently, with any disagreement to be resolved by consensus.

### Data analysis

We examined primarily the proportion of confirmed cases who suffered liver toxicity due to COVID-19 vaccination. This proportion was further classified based on the type of liver pathology induced by the COVID-19 vaccine (i.e., if portal vein thrombosis, autoimmune hepatitis or raised liver enzymes etc.). Descriptive statistics were used to describe the data. For continuous variables, mean and standard deviation were used to summarize the data; and for categorical variables, frequencies and percentages were reported. Microsoft Excel 2019 (Microsoft Corp., Redmond, USA) was used for all statistical analyses.

## Results

### Study characteristics and quality

**Fig. 1:**
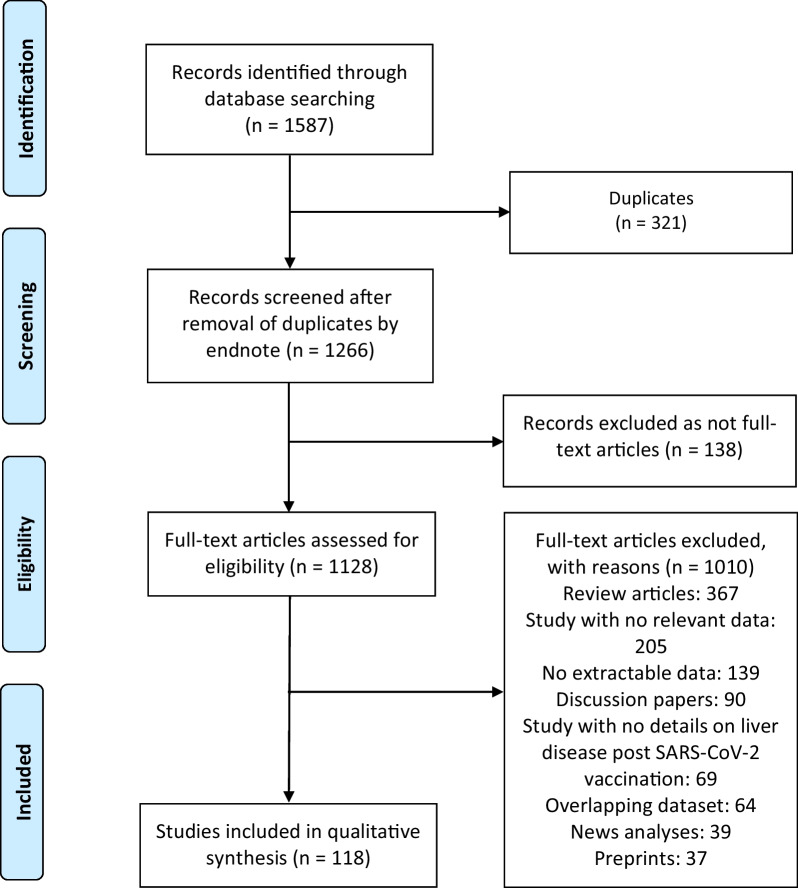
Flow diagram of literature search and data extraction from studies included in the systematic review

A total of 1587 publications were identified (Fig. 1). After exclusion of duplicates and articles that did not fulfil the study inclusion criteria, one hundred and eighteen articles were included in the qualitative synthesis of this systematic review. The reports of two hundred and seventy-five cases identified from these articles are presented by groups based on confirmed diagnoses, laboratory, biopsy and imaging findings [3–13, 23–128]. The detailed characteristics of the included studies are shown in Table 1. There were 107 case report [3–12, 23–41, 43–47, 49–51, 55, 57–59, 61–63, 65–68, 70–125, 127, 128], and 11 case series [13, 42, 48, 52–54, 56, 60, 64, 69, 126] studies. These studies were conducted in United States (n = 20), Italy (n = 15), Germany (n = 10), United Kingdom (n = 9), Japan (n = 6), India (n = 5), Spain (n = 4), Saudi Arabia (n = 4), France (n = 4), Austria (n = 3), Switzerland (n = 4), Iran (n = 4), Republic of Korea (n = 3), Turkey (n = 2), Ireland (n = 2), Portugal (n = 2), Greece (n = 2), The Netherlands (n = 2), Denmark (n = 2), Singapore (n = 2), Brazil (n = 1), Oman (n = 1), Colombia (n = 1), China (n = 1), Israel (n = 1), Taiwan (n = 1), Kuwait (n = 1), Norway (n = 1), Mexico (n = 1), Malaysia (n = 1), Thailand (n = 1), Democratic Republic of the Congo (n = 1), and Australia (n = 1). Only two studies were made within multi-countries (n = 2) [60, 126]. The majority of the studies were single centre [3–12, 23–41, 43–51, 55–59, 61–63, 65–125, 127, 128] and only 8 studies were multi-centre [13, 42, 52–54, 60, 64, 126]. All case reports and case-series studies were assessed for bias using the modified NOS. Thirty-two studies were deemed to have high methodological quality, 83 moderate methodological quality, and 3 low methodological quality; Table 1.

**Table 1:** Summary of the characteristics of the included studies with evidence on new-onset and relapsed liver diseases post-COVID-19 vaccination (n = 118 studies), 2021–2022

Author, year, study location	Study design, setting	Age (years)^a^	Male, n (%)	Ethnicity^b^	Time to presentation from day of vaccination (days)	Comorbidities, n	Vaccine brand and dose	New onset or relapse	Clinical presentation	Laboratory findings	Biopsy findings^c^	Imaging	Treatment received, n	Modified NOS score; and treatment outcome
*Acute cellular rejection of the liver*														
Hughes et al. 2022 [34], United States	Retrospective case report, single centre	65	1 (100)	1 White (Caucasian)	2	1 Cryptogenic cirrhosis 1 Liver transplant recipient 1 Coronary artery disease 1 Diabetes mellitus 1 Hyperlipidaemia	Pfizer-BioNTech, dose 1 [n = 1]	New-onset [n = 1]	1 Extremity weakness 1 Paraesthesia ascending to bilateral hands 1 Hyporeflexia 1 Loss of pinprick sensation 1 Difficulty with walking 1 Bilateral cranial nerve 7 palsies 1 Acute inflammatory demyelinating polyneuropathy	1 Raised liver enzymes 1 Raised bilirubin 1 Thrombocytopenia 1 Raised white blood cells 1 High CRP	Mild acute rejection in his graft	Innumerable new bilobar lesions [n = 1]	1 IVIG 1 Steroid	(NOS, moderate) 1 survived
Sarwar et al. 2022 [69], United States	Retrospective case-series, single centre	Median (IQR), 54 (51–66)	4 (80)	5 White (Caucasian)	Mean (SD), 11.6 (4.6)	5 Liver transplant recipients 3 Non-alcoholic steatohepatitis-related cirrhosis 2 Alcohol-related cirrhosis 2 History of acute cellular rejection	Moderna, dose 1 and dose 2 [n = 3] Pfizer-BioNTech, dose 1 and dose 2 [n = 2]	New-onset [n = 3] Relapsed [n = 2]	Not reported [n = 5]	3 Raised liver enzymes 4 Raised bilirubin	Typical features of T cell-mediated ACRL including portal inflammation of predominantly mixed activated lymphocytes, portal vein phlebitis and bile duct injuries [n = 5]	Not performed [n = 5]	9 Steroid 1 Everolimus 2 Tacrolimus 1 Cyclosporine 1 Mycophenolate mofetil	(NOS, moderate) 5 survived
Valsecchi et al. 2022 [29], Italy	Retrospective case report, single centre	58	0 (0)	1 White (Caucasian)	44	1 Autoimmune cirrhosis 1 Grade II encephalopathy 1 Refractory ascites 1 End-stage liver disease 1 Liver transplant recipient	Pfizer-BioNTech, dose 1 [n = 1]	New-onset [n = 1]	1 Worsened neurologic status 1 Vaccine-induced immune thrombotic thrombocytopenia 1 Graft-versus-host disorder 1 Transplantation-mediated alloimmune thrombocytopenia	1 Low Hb 1 Thrombocytopenia 1 High INR 1 High D-dimer 1 Raised liver enzymes 1 Positive for antibodies directed against (PF4) antibodies	Not performed [n = 1]	Small millimetric high density area on the occipital lobe [n = 1]	1 Heparin 1 Fondaparinux 1 IVIG 1 Steroid	(NOS, moderate) 1 survived
Vyhmeister et al. 2021 [82], United States	Retrospective case report, single centre	64	0 (0)	1 White (Caucasian)	11	1 Cirrhosis 1 Hepatitis C virus 1 Hepatocellular carcinoma 1 Liver transplant recipient	Moderna, dose 1 [n = 1]	New-onset [n = 1]	1 Dark urine 1 Fatigue 1 Malaise	1 Raised liver enzymes	Typical features of ACRL including mixed portal inflammation, bile duct injury, and endotheliitis [n = 1]	Unremarkable [n = 1]	1 Steroid 1 Azathioprine 1 Mycophenolate mofetil 1 Anti-thymocyte globulin	(NOS, moderate) 1 survived
*Acute hepatic failure*														
Barary et al. 2022 [128], Iran	Retrospective case report, single centre	35	1 (100)	1 Persian	8	1 Psychological problems	Oxford Uni-AstraZeneca, dose 1 [n = 1]	New-onset [n = 1]	1 Generalized weakness 1 Abdominal pain 1 Jaundice 1 Fever 1 Headache 1 Vomiting 1 Loss of appetite	1 High D-dimer 1 Thrombocytopenia 1 Low fibrinogen 1 Raised liver enzymes 1 Raised bilirubin 1 DIC 1 High INR	Not performed [n = 1]	Grade I fatty liver disease [n = 1] Mild effusion in subdiaphragmatic space [n = 1]	1 Steroid 1 IVIG 1 Rivaroxaban	(NOS, moderate) 1 died
Efe et al. 2022 [45], Turkey	Retrospective case report, single centre	53	1 (100)	1 White (Caucasian)	10	1 No medical history	Pfizer-BioNTech, dose 1 [n = 1]	New-onset [n = 1]	1 Abdominal pain 1 Erythematous skin eruption 1 Pruritus 1 Hypersensitivity reaction 1 Myalgia 1 Fatigue 1 Jaundice 1 Vaccine-induced immune-mediated liver injury 1 Hepatic encephalopathy 1 Fulminant liver failure	1 Raised liver enzymes 1 Raised bilirubin 1 High INR 1 Elevated IgG	Portal inflammation with interface activity and significant lobular necroinflammatory activity, hepatocellular rosette formation and emperipolesis [n = 1]	Not performed [n = 1]	1 Antihistamines 1 Steroid 1 Plasma exchange 1 Liver transplantation	(NOS, high) 1 survived
Hieber et al. 2022 [35], Germany	Retrospective case report, single centre	24	0 (0)	1 White (Caucasian)	10	1 No medical history	Pfizer-BioNTech, dose 1 [n = 1]	New-onset [n = 1]	1 Fever 1 Fatigue 1 Chills 1 Weakness 1 Nausea 1 Painful cervical and supraclavicular bilateral lymphadenopathy 1 Hemophagocytic lymphohistiocytosis 1 Acute liver failure	1 Reduced white blood cells 1 Raised liver enzymes 1 High LDH 1 Positive ANAs 1 High ferritin	Unremarkable [n = 1]	Splenomegaly [n = 1] Enlarged cervical and supraclavicular lymph nodes [n = 1]	1 Steroid 1 IVIG 1 Anakinra	(NOS, moderate) 1 survived
Sohrabi et al. 2022 [78], Iran	Retrospective case report, single centre	34	1 (100)	1 Persian	1	1 No medical history	Oxford Uni-AstraZeneca, dose 1 [n = 1]	New-onset [n = 1]	1 Headache 1 Nausea 1 Dizziness 1 Abdominal pain 1 Myalgia 1 Yellow eyes 1 Petechiae 1 Gastrointestinal haemorrhage 1 DIC 1 Acute hepatic failure	1 Raised liver enzymes 1 Raised bilirubin 1 High D-dimer 1 High PT 1 High INR 1 Raised white blood cells 1 High APTT 1 High CRP	Liver massive infarction [n = 1]	Massive emboli in portal-vein to the splenic with blockage of the hepatic artery by a thrombus [n = 1]	1 Steroid 1 Antibiotics 1 PRBCs	(NOS, moderate) 1 died
Acute liver injury														
Alqarni et al. 2021 [113], Saudi Arabia	Retrospective case report, single centre	14	0 (0)	1 Arab	3	1 No medical history	Pfizer-BioNTech, dose 2 [n = 1]	New-onset [n = 1]	1 Epigastric pain 1 Epigastric tenderness 1 Diarrhea 1 Nausea 1 Vomiting 1 Jaundice	1 Leukopenia 1 Neutropenia 1 Lymphopenia 1 High PT 1 High APTT 1 High INR	Not performed [n = 1]	Minimal rim of free fluid in the pelvic cavity [n = 1]	1 IV fluids 1 N-acetylcysteine 1 Lactulose 1 Vitamin K 1 Intubation	(NOS, low) 1 survived
Dumortier 2021 [99], France	Retrospective case report, single centre	46	0 (0)	1 White (Caucasian)	12	1 Alcohol-associated liver disease 1 Liver transplant recipient	Pfizer-BioNTech, dose 1 [n = 1]	New-onset [n = 1]	Not reported [n = 1]	1 Raised liver enzymes 1 Raised bilirubin	Not performed [n = 1]	Unremarkable [n = 1]	No treatment [n = 1]	(NOS, moderate) 1 survived
Ghorbani et al. 2022 [44], Iran	Retrospective case report, single centre	62	1 (100)	1 Persian	3	1 Hypertension 1 Diabetes mellitus	Sinopharm COVID-19 vaccine, dose 2 [n = 1]	New-onset [n = 1]	1 Weakness 1 Jaundice 1 Weight loss 1 Itching 1 Yellow eyes 1 Yellow skin	1 Raised liver enzymes 1 Raised bilirubin	Not performed [n = 1]	Hepatitis pattern of injury [n = 1] Portal and lobular inflammation and marked eosinophils infiltration [n = 1]	1 Ursodeoxycholic acid	(NOS, moderate) 1 survived
Kawasaki et al. 2022 [122], Japan	Retrospective case report, single centre	15	0 (0)	1 Asian	1	1 No medical history	Pfizer-BioNTech, dose 1 [n = 1]	New-onset [n = 1]	1 Fever 1 Headache	1 Raised liver enzymes 1 Leukopenia 1 Thrombocytopenia 1 High LDH	Not performed [n = 1]	Unremarkable [n = 1]	1 IV fluids	(NOS, moderate) 1 survived
Mann et al. 2021 [12], United States	Retrospective case report, single centre	61	0 (0)	1 White (Caucasian)	9	1 Irritable bowel disease 1 Cholecystectomy	Pfizer-BioNTech, dose 2 [n = 1]	New-onset [n = 1]	1 Generalized weakness 1 Pain 1 Vomiting 1 Yellow eyes 1 Abdominal tenderness 1 Tachycardia	1 Raised liver enzymes 1 Raised bilirubin 1 Raised white blood cells	Minimal pallor suggesting slight oedema along with scattered inflammatory cells [n = 1]	Increased echogenicity within the liver compatible with fatty infiltrates [n = 1]	1 Antibiotics	(NOS, moderate) 1 survived
Shroff et al. 2021 [13], United States	Retrospective case-series, multicenter	Median (IQR), 63 (49.2–69.5)	6 (37.5)	Not reported	Mean (SD), 25.9 (12.3)	6 Chronic liver disease 4 AIH 3 Cirrhosis 1 Hepatitis C virus 1 Drug-induced liver injury	Pfizer-BioNTech, dose 1 and dose 2 [n = 12] Moderna, dose 1 and dose 2 [n = 4]	New-onset [n = 11] Relapsed [n = 5]	16 Liver injuries 3 Acute liver injuries 1 Primary sclerosing cholangitis	16 Raised liver enzymes 12 Raised bilirubin 7 High INR 5 Positive ANAs 4 Positive ASMAs 1 Elevated IgG	Histopathological findings consistent with AIH [n = 1] Portal inflammation [n = 10] Severe cholestasis [n = 1] Not performed [n = 6]	New severe sclerosing cholangitis [n = 1] Hepatic steatosis [n = 1] Solitary HCC [n = 1] Unremarkable [n = 2] Not performed [n = 2]	8 Steroid 2 N-acetylcysteine 1 Biliary dilatation	(NOS, high) 16 survived
*Autoimmune hepatitis*														
Avci et al. 2021 [112], Turkey	Retrospective case report, single centre	61	0 (0)	1 White (Caucasian)	30	1 Hashimoto’s thyroiditis 1 Hypertension	Pfizer-BioNTech, dose 1 [n = 1]	New-onset [n = 1]	1 Malaise 1 Fatigue 1 Anorexia 1 Nausea 1 Yellow eyes 1 Jaundice	1 Raised liver enzymes 1 Raised bilirubin 1 Positive ANAs 1 Positive ASMAs 1 Elevated IgG	Histopathological findings consistent with AIH [n = 1]	gallbladder was filled with many millimetric stones [n = 1]	1 Steroid 1 Azathioprine	(NOS, moderate) 1 survived
Boettler et al. 2022 [127], Germany	Retrospective case report, single centre	52	1 (100)	1 White (Caucasian)	14	1 Hypothyroidism	Pfizer-BioNTech, dose 1 and dose 2 [n = 1]	New-onset [n = 1]	1 Acute mixed hepatocellular/cholestatic hepatitis [after 1^st^ dose] 1 Severe hepatitis [after 2^nd^ dose] 1 Pruritus 1 Nausea 1 Fatigue 1 Loss of appetite 1 Jaundice 1 Fatigue	1 Highly activated cytotoxic CD8 T-cell infiltrate 1 Raised liver enzymes	Histopathological findings consistent with AIH [n = 1]	Infiltrates consisting of T-cells, macrophages, B-cells, plasma cells and granulocytes in the liver [n = 1]	1 Steroid 1 Ursodeoxycholic acid	(NOS, moderate) 1 survived
Bril et al. 2021 [6], United States	Retrospective case report, single centre	35	0 (0)	1 White (Caucasian)	7	1 Pregnancy 1 Hypertension	Pfizer-BioNTech, dose 1 [n = 1]	New-onset [n = 1]	1 Pruritus 1 Dark urine 1 Jaundice 1 Hepatomegaly	1 Raised liver enzymes 1 Raised bilirubin 1 Raised ammonium 1 Positive ANAs 1 Positive ds-DNA antibodies	Histopathological findings consistent with AIH [n = 1]	Unremarkable [n = 1]	1 Steroid	(NOS, high) 1 survived
Camacho-Domínguez et al. 2022 [37], Colombia	Retrospective case report, single centre	79	1 (100)	1 Hispanic	15	1 Not reported	Oxford Uni-AstraZeneca, dose 1 [n = 1]	New-onset [n = 1]	1 Abdominal pain 1 Jaundice 1 Pruritus 1 Acholia 1 Choluria 1 Yellow skin 1 Abdominal tenderness 1 Esophagitis 1 Gastritis	1 Raised liver enzymes 1 Raised bilirubin 1 Lymphopenia 1 Elevated IgG 1 Positive ANAs 1 Positive ASMAs	Histopathological findings consistent with AIH [n = 1]	Edema of the gallbladder walls with a pattern described in acute hepatitis [n = 1]	1 Steroid 1 Azathioprine	(NOS, moderate) 1 survived
Cao et al. 2021 [110], China	Retrospective case report, single centre	57	0 (0)	1 Asian	2	1 No medical history	Sinovac-CoronaVac, dose 2 [n = 1]	New-onset [n = 1]	1 Dark urine 1 Acholic stools 1 Pruritus 1 Jaundice	1 Raised liver enzymes 1 Raised bilirubin 1 Elevated IgG 1 Positive ANAs 1 Positive anti–Sjögren syndrome antigen A 1 Positive anti–major centromere autoantigen B 1 Positive anti–Sjögren syndrome antigen B	Histopathological findings consistent with AIH [n = 1]	Unremarkable [n = 1]	1 Ursodeoxycholic acid 1 Steroid 1 Azathioprine	(NOS, high) 1 survived
Clayton-Chubb et al. 2021 [101], Australia	Retrospective case report, single centre	36	1 (100)	1 Arab	26	1 Hypertension 1 Laser eye surgery	Oxford Uni-AstraZeneca, dose 1 [n = 1]	New-onset [n = 1]	1 Pruritus	1 Raised liver enzymes 1 Raised bilirubin 1 Positive ANAs 3 Elevated IgG	Histopathological findings consistent with AIH [n = 1]	Mild peri-portal oedema [n = 1]	1 Steroid	(NOS, high) 1 survived
Efe et al. 2022 [126], Multicounty	Retrospective case-series, multicenter	Median (IQR), 48 (18–79)	32 (36.8)	Not reported	Median (IQR), 15 (3–65)	13 Diabetes mellitus 13 Hypertension 12 Pre-existing liver disease 7 NAFLD 1 Primary biliary cholangitis 1 Hepatitis C infection 1 Liver transplant 1 Breast cancer 1 Pemphigus vulgaris 1 Polycythemia vera	Pfizer-BioNTech, dose not reported [n = 51] Moderna, dose not reported [n = 16] Oxford Uni-AstraZeneca, dose not reported [n = 20]	New-onset [n = 48] Not reported [n = 39]	65 Fatigue 55 Nausea 34 Jaundice 21 Abdominal pain 10 Itching 7 Rash 7 Fever	56 Positive ANAs 15 Positive ASMAs 5 Positive AMAs 53 Elevated IgG 1 Anti-SLA 1 Positive LC-1 7 Raised liver enzymes	Histopathological findings consistent with AIH [n = 34]	Not reported [n = 87]	46 Steroid 9 Azathioprine 2 Mycophenolate mofetil 9 Plasma exchange 1 IVIG 1 Liver transplantation	(NOS, moderate) 87 survived
Erard et al. 2021 [99], France	Retrospective case reports, single centre	Median (IQR), 73 (68–73)	0 (0)	3 Whites (Caucasians)	Mean (SD), 17 (6.1)	1 Not reported	Pfizer-BioNTech, dose 2 [n = 1] Moderna, dose 2 [n = 1] Oxford Uni-AstraZeneca, dose 3 [n = 1]	New-onset [n = 3]	2 Fatigue 3 Pruritus 3 Jaundice 1 Hepatic encephalopathy 1 Liver failure 1 Sepsis	3 Raised liver enzymes 3 Raised bilirubin 1 High INR 3 Positive ANAs	Histopathological findings consistent with AIH [n = 3]	Unremarkable [n = 1]	2 Steroid	(NOS, moderate) 2 survived 1 died
Fimiano et al. 2022 [68], Italy	Retrospective case report, single centre	63	0 (0)	1 White (Caucasian)	54	1 Postmenopausal hypothyroidism 1 Family history of 1st-degree relative with coeliac disease	Pfizer-BioNTech, dose 1 [n = 1]	New-onset [n = 1]	1 Abdominal pain 1 Nausea 1 Hyperchromic urines 1 Jaundice 1 Hypoechoic stools	1 Raised liver enzymes 1 Raised bilirubin 1 Positive ATA 1 Elevated IgG	Histopathological findings consistent with AIH [n = 1]	Unremarkable [n = 1]	1 Steroid 1 Azathioprine	(NOS, moderate) 1 survived
Garrido et al. 2021 [7], Portugal	Retrospective case report, single centre	65	0 (0)	1 White (Caucasian)	14	1 Polycythemia vera	Moderna, dose 1 [n = 1]	New-onset [n = 1]	1 Jaundice 1 Dark urine 1 Abdominal pain 1 Hepatomegaly	1 Raised liver enzymes 1 Raised bilirubin 1 Positive ANAs 3 Elevated IgG	Histopathological findings consistent with AIH [n = 1]	Hepatomegaly [n = 1]	1 Steroid	(NOS, moderate) 1 survived
Ghielmetti et al. 2021 [102], Switzerland	Retrospective case report, single centre	63	1 (100)	1 White (Caucasian)	7	1 Diabetes mellitus 1 Ischemic heart disease	Moderna, dose 1 [n = 1]	New-onset [n = 1]	1 Jaundice 1 Fatigue 1 Anorexia 1 Hepatomegaly	1 Raised liver enzymes 1 Raised bilirubin 1 Positive ANAs 1 Elevated IgG	Histopathological findings consistent with AIH [n = 1]	Unremarkable [n = 1]	1 Steroid	(NOS, moderate) 1 survived
Goulas et al. 2021 [97], Greece	Retrospective case report, single centre	52	0 (0)	1 White (Caucasian)	14	1 No medical history	Moderna, dose 1 [n = 1]	New-onset [n = 1]	1 Malaise 1 Jaundice	1 Raised liver enzymes 1 Raised bilirubin 1 High CRP 1 High ESR 1 Positive ANAs 1 Positive ASMAs 1 Elevated IgG	Histopathological findings consistent with AIH [n = 1]	Unremarkable [n = 1]	1 Steroid 1 Azathioprine	(NOS, moderate) 1 survived
Hasegawa et al. 2022 [124], Japan	Retrospective case report, single centre	82	0 (0)	1 Asian	7	1 Hepatitis C infection	Pfizer-BioNTech, dose 1 [n = 1]	New-onset [n = 1]	1 Fatigue 1 Loss of appetite 1 Severe liver injury	1 Positive ANAs 1 Elevated IgG 1 Raised liver enzymes 1 Raised bilirubin	Histopathological findings consistent with AIH [n = 1]	Unremarkable [n = 1]	1 Steroid	(NOS, moderate) 1 survived
Kang et al. 2022 [123], Republic of Korea	Retrospective case report, single centre	27	0 (0)	1 Asian	14	1 No medical history	Pfizer-BioNTech, dose 2 [n = 1]	New-onset [n = 1]	1 Jaundice 1 Hepatomegaly 1 Nausea 1 Vomiting 1 Headache 1 Fever 1 Dark urine 1 Enteritis 1 Diarrhea	1 Elevated IgG 1 Raised liver enzymes 1 Raised bilirubin 1 Positive ANAs	Histopathological findings consistent with AIH [n = 1]	Splenomegaly [n = 1] Gallbladder wall thickening [n = 1]	1 Steroid	(NOS, moderate) 1 survived
Lasagna et al. 2022 [120], Italy	Retrospective case report, single centre	52	0 (0)	1 White (Caucasian)	10	1 Lung adenocarcinoma with bone metastases 1 Hepatitis B infection	Pfizer-BioNTech, dose 1 [n = 1]	New-onset [n = 1]	1 Hepatitis 1 Colitis 1 Diarrhea	1 Raised liver enzymes 1 High LDH 1 Elevated IgG	Histopathological findings consistent with AIH [n = 1]	Unremarkable [n = 1]	1 Steroid	(NOS, moderate) 1 survived
Lee et al. 2022 [119], Republic of Korea	Retrospective case report, single centre	57	0 (0)	1 Asian	14	1 No medical history	Pfizer-BioNTech, dose 1 [n = 1]	New-onset [n = 1]	1 Weakness 1 Fatigue	1 Raised liver enzymes 1 Positive ANAs 1 Positive AMAs 1 Elevated IgG	Histopathological findings consistent with AIH [n = 1]	Unremarkable [n = 1]	1 Ursodeoxycholic acid	(NOS, moderate) 1 survived
Lodato et al. 2021 [105], Italy	Retrospective case report, single centre	43	0 (0)	1 White (Caucasian)	15	1 Hyperlipidemia	Pfizer-BioNTech, dose 2 [n = 1]	New-onset [n = 1]	1 Jaundice 1 Itching 1 Abdominal pain	1 Raised liver enzymes 1 Raised bilirubin 1 Elevated IgG	Histopathological findings consistent with AIH [n = 1]	Unremarkable [n = 1]	1 Steroid 1 N-acetylcysteine	(NOS, high) 1 survived
Londoño et al. 2021 [108], Spain	Retrospective case report, single centre	41	0 (0)	1 White (Caucasian)	7	1 Premature ovarian failure 1 Substitutive hormonal therapy	Moderna, dose 2 [n = 1]	New-onset [n = 1]	1 Epigastric pain 1 Nausea 1 Vomiting 1 Dark urine 1 Jaundice	1 Raised liver enzymes 1 Raised bilirubin 1 Positive ANAs 1 Positive ASMAs 1 Positive LC-1 1 Elevated IgG 1 Anti-SLA	Histopathological findings consistent with AIH [n = 1]	Unremarkable [n = 1]	1 Steroid	(NOS, high) 1 survived
Mahalingham et al. 2022 [43], United Kingdom	Retrospective case report, single centre	32	0 (0)	1 White (Caucasian)	21	1 Liver transplant recipient 1 Autoimmune hepatitis	Pfizer-BioNTech, dose 3 [n = 1]	Relapsed [n = 1]	Asymptomatic	1 Raised liver enzymes	Histopathological findings consistent with AIH [n = 1]	Unremarkable [n = 1]	1 Steroid 1 Azathioprine	(NOS, moderate) 1 survived
McShane et al. 2021 [107], Ireland	Retrospective case report, single centre	71	0 (0)	1 White (Caucasian)	4	1 Cholecystectomy 1 Left total hip replacement 1 Osteoarthritis of the knees	Moderna, dose 1 [n = 1]	New-onset [n = 1]	1 Jaundice	1 Raised liver enzymes 1 Raised bilirubin 1 Elevated IgG 1 Positive ASMAs	Histopathological findings consistent with AIH [n = 1]	Distal common bile duct dilation consistent with prior cholecystectomy [n = 1]	1 Steroid	(NOS, high) 1 survived
Mekritthikrai et al. 2022 [118], Thailand	Retrospective case report, single centre	52	0 (0)	1 Asian	7	1 Hypertension 1 Dyslipidemia	Sinovac-CoronaVac, dose 2 [n = 1]	New-onset [n = 1]	1 Jaundice 1 Fatigue 1 Yellow eyes	1 Raised liver enzymes 1 Raised bilirubin 1 Positive ANAs 1 Positive ASMAs 1 Elevated IgG	Histopathological findings consistent with AIH [n = 1]	Liver cirrhosis [n = 1]	1 Steroid 1 Azathioprine	(NOS, high) 1 survived
Nyein et al. 2022 [117], Singapore	Retrospective case report, single centre	34	1 (100)	1 Asian	14	1 No medical history	Moderna, dose 1 [n = 1]	New-onset [n = 1]	1 Pruritus 1 Fever 1 Jaundice	1 Raised liver enzymes 1 Raised bilirubin 1 Elevated IgG 1 Positive ANAs 1 Positive AMAs 1 Acute hepatitis 1 Acute cholestasis	Histopathological findings consistent with AIH [n = 1]	Unremarkable [n = 1]	1 Ursodeoxycholic acid	(NOS, high) 1 survived
Palla et al. 2022 [87], Greece	Retrospective case report, single centre	40	0 (0)	1 White (Caucasian)	30	1 Sarcoidosis	Pfizer-BioNTech, dose 2 [n = 1]	New-onset [n = 1]	Asymptomatic	1 Raised liver enzymes 1 Positive ANAs 1 Elevated IgG	Histopathological findings consistent with AIH [n = 1]	Unremarkable [n = 1]	1 Steroid	(NOS, high) 1 survived
Rela et al. 2021 [104], India	Retrospective case reports, single centre	38 and 65	1 (50)	2 Indians	Mean (SD), 18 (2.8)	1 Hypothyroidism 1 Diabetes mellitus 1 Jaundice	Covishield, dose 1 [n = 2]	New-onset [n = 1] Relapsed [n = 1]	2 Fever 1 Anorexia 1 Fatigue 2 Jaundice 1 Altered sensorium 1 Leg edema 1 Dark urine	2 Raised liver enzymes 2 Raised bilirubin 2 High INR 1 Elevated IgG 1 Positive ANAs	Histopathological findings consistent with AIH [n = 2]	Unremarkable [n = 1] Hepatomegaly [n = 1] Inter-bowel free fluid [n = 1]	2 Steroid 1 Exchange transfusion	(NOS, moderate) 1 survived 1 died
Rigamonti et al. 2022 [42], Italy	Retrospective case-series, multicenter	Median (IQR), 62 (32–80)	6 (50)	12 Whites (Caucasians)	48 for [dose 1] 10 for [dose 2]	3 Thyroiditis 2 Rheumatoid arthritis 1 Systemic lupus erythematosus	Pfizer-BioNTech, dose not reported [n = 7] Moderna, dose not reported [n = 2] Oxford Uni-AstraZeneca, dose not reported [n = 3]	Not reported [n = 12]	8 Jaundice	10 Raised liver enzymes 8 Raised bilirubin 6 Positive ANAs 1 Positive ASMAs 1 Liver/kidney microsome type 1 antibodies	Histopathological findings consistent with AIH [n = 11]	Not reported [n = 12]	Not reported [n = 12]	(NOS, moderate) 12 outcome was not reported
Rocco et al. 2021 [106], Italy	Retrospective case report, single centre	80	0 (0)	1 White (Caucasian)	7	1 Hyperlipidemia 1 Hashimoto’s thyroiditis 1 Acute glomerulonephritis	Pfizer-BioNTech, dose 2 [n = 1]	New-onset [n = 1]	1 Jaundice 1 Dark urine	1 Raised liver enzymes 1 Raised bilirubin 1 Positive ANAs 1 Elevated IgG	Histopathological findings consistent with AIH [n = 1]	Enlarged reactive hilar lymph nodes [n = 1]	1 Steroid	(NOS, high) 1 survived
Romero-Salazar et al. 2022 [41], Spain	Retrospective case report, single centre	76	1 (100)	1 White (Caucasian)	Not reported	1 Liver cirrhosis 1 Primary biliary cholangitis	Pfizer-BioNTech, dose 3 [n = 1]	New-onset [n = 1]	Not reported [n = 1]	1 Raised liver enzymes 1 Raised bilirubin 1 Elevated IgG 1 Positive ANAs	Histopathological findings consistent with AIH [n = 1]	Not reported [n = 1]	1 Ursodeoxycholic acid 1 Obeticholic acid 1 Steroid 1 Azathioprine	(NOS, moderate) 1 survived
Shahrani et al. 2022 [115], Malaysia	Retrospective case reports, single center	Median (IQR), 63 (59–63)	0 (0)	3 Asians	Median (IQR), 12 (10–12)	1 Dyslipidemia 1 Ulcerative colitis 1 Primary sclerosing cholangitis 1 No medical history	Oxford Uni-AstraZeneca, dose 2 [n = 2] Pfizer-BioNTech, dose 3 [n = 1]	New-onset [n = 3]	3 Jaundice	3 Raised liver enzymes 3 Raised bilirubin 3 Elevated IgG 1 Positive ANAs 1 Positive AMAs	Histopathological findings consistent with AIH [n = 1]	Unremarkable [n = 3]	3 Steroid	(NOS, high) 2 survived 1 died
Suzuki et al. 2021 [84], Japan	Retrospective case reports, single centre	Median (IQR), 78 (75–78)	0 (0)	3 Asians	Median (IQR), 7 (4–7)	1 Gastroesophageal reflux esophagitis 1 Hyperlipidemia 1 Primary biliary cholangitis	Pfizer-BioNTech, dose 2 [n = 2] Pfizer-BioNTech, dose 1 [n = 1]	New-onset [n = 3]	1 Jaundice 1 Dark urine 1 Fever 1 Malaise	3 Liver injury 3 Raised liver enzymes 3 Raised bilirubin 3 Positive ANAs 3 Elevated IgG 2 High INR	Histopathological findings consistent with AIH [n = 3]	Peripheral edema [n = 2]	3 Steroid	(NOS, high) 3 survived
Tan et al. 2021 [8], Singapore	Retrospective case report, single centre	56	0 (0)	1 Asian	42	1 Hyperlipidemia	Moderna, dose 1 [n = 1]	New-onset [n = 1]	1 Anorexia 1 Jaundice 1 Yellow eyes	1 Raised liver enzymes 1 Raised bilirubin 1 Elevated IgG 1 Positive ANAs 1 Positive ASMAs	Histopathological findings consistent with AIH [n = 1]	Unremarkable [n = 1]	1 Steroid	(NOS, high) 1 survived
Torrente et al. 2021 [86], Spain	Retrospective case report, single centre	46	0 (0)	1 White (Caucasian)	21	1 Hypothyroidism 1 Hypertransaminasemia 1 Anaemia	Oxford Uni-AstraZeneca, dose 1 [n = 1]	Relapsed [n = 1]	Asymptomatic	1 Raised liver enzymes 1 Low Hb 1 Positive ANAs 1 Low ferritin 1 Positive HLA-DRB1*03 and 04 1 Positive HLA DQ2 and DQ8 1 Elevated IgG	Histopathological findings consistent with AIH [n = 1]	Unremarkable [n = 1]	1 Steroid 1 Azathioprine	(NOS, moderate) 1 survived
Tun et al. 2021 [85], United Kingdom	Retrospective case report, single centre	47	1 (100)	1 White (Caucasian)	3 for [dose 1] 18 for [dose 2]	1 No medical history	Moderna, dose 1 and dose 2 [n = 1]	New-onset [n = 1]	1 Malaise 1 Jaundice 1 Hepatomegaly	1 Raised liver enzymes 1 Raised bilirubin 1 Positive ANAs 1 Elevated IgM 1 Elevated IgG 1 High PT	Histopathological findings consistent with AIH [n = 1]	Unremarkable [n = 1]	1 Steroid	(NOS, high) 1 survived
Vuille-Lessard et al. 2021 [103], Switzerland	Retrospective case report, single centre	76	0 (0)	1 White (Caucasian)	2	1 Hashimoto’s thyroiditis 1 Urothelial carcinoma 1 Low blood pressure	Moderna, dose 1 [n = 1]	New-onset [n = 1]	1 Dark urine 1 Weight loss 1 Fatigue 1 Yellow eyes 1 Hepatomegaly	1 Raised liver enzymes 1 Raised bilirubin 1 Positive ANAs 1 Positive ASMAs 1 Elevated IgG	Histopathological findings consistent with AIH [n = 1]	Slightly enlarged and hyperechogenic liver [n = 1]	1 Steroid 1 Azathioprine	(NOS, moderate) 1 survived
Zhou et al. 2021 [80], Germany	Retrospective case report, single centre	36	0 (0)	1 White (Caucasian)	11	1 Primary sclerosing cholangitis 1 Ulcerative colitis 1 Pruritus	Moderna, dose 1 [n = 1]	Relapsed [n = 1]	Asymptomatic except for minor muscle aches	1 Raised liver enzymes 1 Raised bilirubin 1 High INR 1 Positive ANAs 1 Positive ds-DNA antibodies 1 Elevated IgG	Histopathological findings consistent with AIH [n = 1]	Unremarkable [n = 1]	1 Steroid 1 Azathioprine	(NOS, high) 1 survived
*Hepatic porphyria*														
Jud et al. 2021 [92], Austria	Retrospective case report, single centre	34	0 (0)	1 White (Caucasian)	4	1 Hashimoto’s thyroiditis 1 Appendectomy	Oxford Uni-AstraZeneca, dose 1 [n = 1]	New-onset [n = 1]	1 Fever 1 Pinprick sensation in her chest and thoracic spine 1 Dizziness 1 Abdominal pain 1 Dark urine 1 SIADH 1 Vomiting 1 Loose stool 1 Pollakisuria 1 dysuria 1 Hypertension 1 Leg dysesthesia	1 Hyponatremia 1 High creatinine 1 Thrombocytopenia 1 High urine porphyrins 1 High urine 5-aminolevulinic acid 1 High urine porphobilinogen	Not performed [n = 1]	Unremarkable [n = 1]	1 Hemin 1 Metamizole 1 Butylscopolamine bromide 1 Crystalloid fluid 1 Antibiotic 1 Piritramide 1 Furosemide 1 Urapidil	(NOS, moderate) 1 survived
*Hepatomegaly*														
Cory et al. 2021 [100], United Kingdom	Retrospective case report, single centre	36	0 (0)	1 White (Caucasian)	9	1 No medical history	Oxford Uni-AstraZeneca, dose 1 [n = 1]	New-onset [n = 1]	1 Abdominal tenderness 1 Pleuritic pain 1 Pericardial rub 1 Hepatomegaly 1 Splenomegaly 1 Pericarditis	1 Thrombocytopenia 1 High ferritin 1 High CRP 1 High LDH	Reactive picture [n = 1]	Hepatomegaly [n = 1] Splenomegaly [n = 1] Pleural effusions [n = 1] Pericarditis [n = 1]	1 Antibiotics 1 Steroid 1 IVIG 1 IV fluids 1 Analgesics	(NOS, low) 1 survived
Manzo et al. 2021 [88], Italy	Retrospective case report, single centre	69	0 (0)	1 White (Caucasian)	1	1 No medical history	Pfizer-BioNTech, dose 1 [n = 1]	New-onset [n = 1]	1 Pain in the shoulder and pelvis 1 Stiffness 1 Fatigue 1 Fever 1 Polymyalgia rheumatica	1 High CRP 1 High ESR	Not performed [n = 1]	Mild hepatomegaly [n = 1]	1 Steroid	(NOS, moderate) 1 survived
Patil and Patil 2021 [24], India	Retrospective case report, single centre	22	0 (0)	1 Indian	10	1 Infective jaundice	Covishield, dose 2 [n = 1]	New-onset [n = 1]	1 Pain in right knee 1 Fever 1 Polyarthralgia 1 Bipedal edema 1 Cutaneous rash over fingertips 1 Petechiae over lower limb 1 Left cervical lymph node 1 Mild liver enlargement 1 Systemic lupus erythematosus	1 Positive ANAs 1 Positive anti-double strand deoxyribonucleic acid 1 Elevated IgG 1 Low Hb 1 Pancytopenia 1 Thrombocytopenia 1 Raised white blood cells 1 High leukocytes 1 High ESR 1 High LDH 1 High D-dimer 1 High APTT	Not performed [n = 1]	Bilateral cervical lymphadenopathy [n = 1] Mild hepatomegaly [n = 1]	1 Steroid 1 HCQ 1 Mycophenolate mofetil 1 Furosemide 1 Telmisartan 1 Folic acid 1 Calcium 1 Vitamin D3	(NOS, moderate) 1 survived
*Jaundice*														
Al Aoun and Motabi 2021 [75], Saudi Arabia	Retrospective case report, single centre	45	0 (0)	1 Arab	3	1 No medical history	Pfizer-BioNTech, dose 1 [n = 1]	New-onset [n = 1]	1 SOB 1 Palpitations 1 Dark urine 1 Fatigue 1 Tachycardia 1 Jaundice 1 Pallor	1 High reticulocyte count 1 Low Hb 1 High LDH 1 Raised bilirubin	Not performed [n = 1]	Unremarkable [n = 1]	1 PRBCs 1 Rituximab	(NOS, moderate) 1 survived
Al-Ahmad et al. 2021 [71], Kuwait	Retrospective case report, single centre	37	1 (100)	1 Arab	10	1 Smoking 1 Polycythemia 1 Venesection	Oxford Uni-AstraZeneca, dose 1 [n = 1]	New-onset [n = 1]	1 Dizziness 1 Fatigue 1 headache 1 SOB 1 Palpitation 1 Acquired haemolytic anaemia 1 Dark urine 1 Tachycardia 1 Jaundice 1 Pallor 1 Purpuric eruptions on extremities	1 Fragmented erythrocytes 1 Thrombocytopenia 1 Low Hb 1 High reticulocyte count 1 Thrombocytopenia 1 High LDH	Not performed [n = 1]	Unremarkable [n = 1]	1 Steroid 1 Rituximab 1 Plasma exchange	(NOS, moderate) 1 survived
Guri et al. 2022 [125], Switzerland	Retrospective case report, single centre	53	1 (100)	1 White (Caucasian)	2	1 Benign recurrent intrahepatic cholestasis 1 Family history of benign recurrent intrahepatic cholestasis	Pfizer-BioNTech, dose 1 [n = 1]	Relapsed [n = 1]	1 Jaundice 1 Pruritus 1 Fever 1 Fatigue 1 Nausea 1 Acute kidney injury	1 Raised liver enzymes 1 Raised bilirubin	Histopathological findings consistent with benign recurrent intrahepatic cholestasis [n = 1]	Not performed [n = 1]	1 Colestyramine 1 Ursodeoxycholic acid 1 Rifampicin 1 Phototherapy	(NOS, high) 1 survived
Lensen et al. 2021 [90], The Netherlands	Retrospective case report, single centre	82	0 (0)	1 White (Caucasian)	3	1 Alzheimer’s disease 1 Hepatitis C infection 1 Hepatitis B infection 1 Diabetes mellitus 1 Hypertension 1 Osteoarthritis 1 Portal hypertension 1 Oesophageal varices 1 Hepatic cirrhosis 1 Thrombocytopenia 1 Allergy to wasp sting	Pfizer-BioNTech, dose 1 [n = 1]	Relapsed [n = 1]	1 Jaundice 1 Somnolence 1 Chills 1 Yellow eyes 1 Decreased consciousness 1 Abdominal pain 1 Coma	1 Raised liver enzymes 1 Raised bilirubin 1 High CRP	Not performed [n = 1]	Not performed [n = 1]	Not reported [n = 1]	(NOS, moderate) 1 died
Peralta-Amaro et al. 2022 [74], Mexico	Retrospective case report, single centre	18	1 (100)	1 Hispanic	22	1 No medical history	Oxford Uni-AstraZeneca, dose 1 [n = 1]	New-onset [n = 1]	1 Fever 1 Headache 1 Diarrhoea 1 Conjunctival injection 1 Skin lesions on the thorax and hands 1 Sudden pain 1 Cyanosis 1 Leg coolness 1 Numbness 1 Rash 1 Palmar erythema with superficial scaling 1 Cracked and erythematous lips 1 Strawberry tongue 1 Jaundice 1 Cervical lymphadenopathy 1 Acute arterial insufficiency of the right foot and leg	1 High CRP 1 Raised liver enzymes 1 Hypoalbuminemia 1 Raised bilirubin 1 High LDH 1 Thrombocytopenia 1 High PT 1 High APTT 1 High leukocytes 1 High creatinine	Not performed [n = 1]	Arterial thrombosis of the right leg [n = 1]	1 IVIG 1 Acetylsalicylic acid	(NOS, high) 1 survived
Pérez‐Lamas et al. 2021 [73], Spain	Retrospective case report, single centre	57	0 (0)	1 White (Caucasian)	2	1 Cold agglutinin disease 1 Anaemia	Pfizer-BioNTech, dose 1 and dose 2 [n = 1]	New-onset [n = 1]	1 Chills 1 Weakness 1 SOB 1 Lumbar pain 1 Jaundice 1 Paleness 1 Autoimmune haemolytic anaemia	1 Hemoglobinuria 1 High reticulocyte count 1 Low Hb 1 Raised bilirubin 1 High ferritin 1 High D-dimer 1 Positive ANAs	Not performed [n = 1]	Not performed [n = 1]	1 Steroid	(NOS, high) 1 survived
Wong et al. 2021 [81], United States	Retrospective case report, single centre	61	0 (0)	1 White (Caucasian)	5	1 Breast cancer	Pfizer-BioNTech, dose 2 [n = 1]	New-onset [n = 1]	1 Generalized cutaneous hypersensitivity reaction 1 Fever 1 Fatigue 1 Generalized myalgia 1 Jaundice 1 Rash 1 Nausea 1 Headache 1 Acute hepatitis	1 Raised liver enzymes 1 Raised bilirubin	Not performed [n = 1]	Unremarkable [n = 1]	1 Steroid	(NOS, moderate) 1 survived
Yoshida et al. 2022 [72], Japan	Retrospective case report, single centre	57	1 (100)	1 Asian	7	1 No medical history	Pfizer-BioNTech, dose 1 [n = 1]	New-onset [n = 1]	1 Anorexia 1 Fatigue 1 Jaundice 1 Acquired haemolytic anaemia 1 Anaphylactic shock 1 Respiratory distress	1 Fragmented erythrocytes 1 Thrombocytopenia 1 ITP 1 Acute hepatitis 1 Low Hb 1 Raised white blood cells 1 High reticulocyte count 1 Raised liver enzymes 1 Raised bilirubin 1 High creatinine	Not performed [n = 1]	Unremarkable [n = 1]	1 Steroid 1 Rituximab 1 Plasma exchange 1 FFP 1 Epinephrine	(NOS, moderate) 1 survived
*Portal vein thrombosis*														
Aladdin et al. 2021 [67], Saudi Arabia	Retrospective case report, single centre	36	0 (0)	1 Arab	14	1 Diabetes mellitus	Oxford Uni-AstraZeneca, dose 1 [n = 1]	New-onset [n = 1]	1 Convulsions 1 Weakness 1 Fever 1 Vomiting 1 Headache 1 Tachycardia 1 Brisk deep tendon reflexes 1 Babinski sign 1 Hypotension 1 DIC 1 Acute kidney injury 1 Lactic acidosis 1 Multi-organ failure 1 Cardiac arrest 1 Worsening of the neurological state	1 Low Hb 1 Raised white blood cells 1 Raised liver enzymes 1 High D-dimer 1 High INR 1 High PT 1 High APTT 1 Thrombocytopenia 1 Low fibrinogen 1 High creatinine	Not performed [n = 1]	Extensive portal vein thrombosis [n = 1] Superior mesenteric vein thrombosis [n = 1] Splenic and hepatic infarction [n = 1]	1 Heparin 1 Antibiotics 1 Antivirals 1 Intubation 1 MV 1 Ionotropic support 1 PRBCs 1 Hemodialysis	(NOS, moderate) 1 died
Asif et al. 2021 [66], United States	Retrospective case report, single centre	28	1 (100)	1 White (Caucasian)	10	1 No medical history	Johnson & Johnson COVID-19 vaccine, dose 1 [n = 1]	New-onset [n = 1]	1 Headache 1 Nausea 1 Vision changes 1 Photophobia 1 Cerebral venous sinus thrombosis 1 Pulmonary emboli	1 Thrombocytopenia 1 Positive for antibodies directed against (PF4) antibodies 1 High D-dimer 1 Positive heparin-induced thrombocytopenia	Not performed [n = 1]	Multiple acute pulmonary emboli [n = 1] Right hepatic vein thrombosis [n = 1]	1 Anticoagulant 1 IVIG 1 Argatroban 1 Acetazolamide 1 Apixaban	(NOS, moderate) 1 survived
Asmat et al. 2021 [65], United Kingdom	Retrospective case report, single centre	47	0 (0)	1 White (Caucasian)	10	1 Migraine	Oxford Uni-AstraZeneca, dose 1 [n = 1]	New-onset [n = 1]	1 Headache 1 Photophobia 1 Periorbital pain 1 Neck stiffness 1 Back pain 1 Vaccine-associated thrombocytopenia 1 Heparin-induced thrombocytopenia 1 Abdominal pain 1 Chest pain	1 Thrombocytopenia 1 Raised liver enzymes 1 High D-dimer 1 Positive for antibodies directed against (PF4) antibodies 1 Positive heparin-induced thrombocytopenia	Not performed [n = 1]	Pulmonary embolism [n = 1] Completely occluded portal vein [n = 1] Acute thrombosis extending into the superior mesenteric vein and splenic vein [n = 1]	1 Sumatriptan 1 IVIG 1 Fondaparinux 1 Apixaban	(NOS, moderate) 1 survived
Bersinger et al. 2021 [111], France	Retrospective case report, single centre	21	0 (0)	1 White (Caucasian)	9	1 Migraine 1 Smoking 1 Contraception	Oxford Uni-AstraZeneca, dose 1 [n = 1]	New-onset [n = 1]	1 Headache 1 Seizure 1 Fall 1 Dislocation of right knee 1 Right-sided hemiplegia 1 Expressive aphasia	1 Thrombocytopenia 1 Positive for antibodies directed against (PF4) antibodies 1 Positive sensitized serotonin release assay	Not performed [n = 1]	Thrombosis in the deep and superficial cerebral veins [n = 1] Thrombosis of the left jugular vein [n = 1] Left frontoparietal venous haemorrhagic infarction [n = 1] Pulmonary embolism [n = 1] Hepatic and external iliac venous thrombosis [n = 1]	1 Heparin 1 Intubation 1 MV 1 Sedation 1 Anticoagulant 1 IVIG 1 Fondaparinux 1 Cranioplasty	(NOS, moderate) 1 survived
Centonze et al. 2021 [109], Italy	Retrospective case report, single centre	32	0 (0)	1 White (Caucasian)	11	1 DBD donor	Oxford Uni-AstraZeneca, dose 1 [n = 1]	New-onset [n = 1]	Not reported [n = 1]	1 Thrombocytopenia 1 High D-dimer 1 High APTT 1 Low fibrinogen	Not performed [n = 1]	Hepatic veins thrombosis [n = 1] Cerebral venous sinus thrombosis [n = 1]	Not reported [n = 1]	(NOS, moderate) 1 died
Ciccone et al. 2021 [64], Italy	Retrospective case-series, multicenter	Median (IQR), 48 (36.7–54.7)	0 (0)	4 Whites (Caucasians)	Mean (SD), 3.7 (2.6)	1 Factor II mutation 1 Contraception 2 No medical history	Oxford Uni-AstraZeneca, dose 1 [n = 4]	New-onset [n = 4]	2 Fever 4 Headache 1 Nausea 2 Vomiting	4 High D-dimer 3 High INR 4 Thrombocytopenia	Not performed [n = 1]	Suprahepatic vein thrombosis [n = 1] Portal and mesenteric veins thrombosis [n = 1] Aortic arch, thoracic aorta, portal, suprahepatic, right coronary, pulmonary and basilar arteries thrombosis [n = 1] Pulmonary thromboembolism, portal vein and inferior cava thrombosis [n = 1]	3 Heparin 3 Mannitol 1 Thrombectomy 2 Craniectomy 3 Steroid 1 Plasmapheresis 1 Fresh frozen plasma 2 Fondaparinux	(NOS, high) 3 in a coma 1 died
Curcio et al. 2022 [63], Italy	Retrospective case report, single centre	68	1 (100)	1 White (Caucasian)	13	1 Hypertension 1 Euthyroid nodular goitre	Johnson & Johnson COVID-19 vaccine, dose 1 [n = 1]	New-onset [n = 1]	1 Leg edema 1 Leg pain 1 Weakness 1 Dizziness 1 Dyspnoea 1 Tachypnea	1 Thrombocytopenia 1 High D-dimer 1 High LDH 1 High CRP 1 Low Hb 1 High INR 1 Positive for antibodies directed against (PF4) antibodies	Not performed [n = 1]	Massive bilateral pulmonary artery embolism thrombosis [n = 1] Right intrahepatic portal thrombosis [n = 1]	1 Steroid 1 IVIG 1 Anticoagulant 1 Implanted inferior caval vein filter 1 Fondaparinux	(NOS, moderate) 1 survived
D'agostino et al. 2021 [62], Italy	Retrospective case report, single centre	54	0 (0)	1 White (Caucasian)	12	1 Not reported	Oxford Uni-AstraZeneca, dose 1 [n = 1]	New-onset [n = 1]	1 DIC 1 Acute cerebrovascular accident 1 Worsening of the neurological state	1 Thrombocytopenia 1 Low Hb 1 High PT 1 High D-dimer 1 High APTT	Not performed [n = 1]	Filling defects at the level of left portal branch and at the level of right suprahepatic vein [n = 1]	1 Plain old balloon angioplasty of the right coronary artery was performed 1 Antiplatelet	(NOS, high) 1 died
De Michele et al. 2021 [61], Italy	Retrospective case reports, single centre	57 and 55	0 (0)	2 Whites (Caucasians)	7 and 9	2 Hypothyroidism 1 Breast cancer 1 Left-sided hemiplegia 1 Gaze deviation 1 Dysarthria 1 Left neglect	Oxford Uni-AstraZeneca, dose 1 [n = 2]	New-onset [n = 2]	1 Worsening of the neurological state 1 ARDS 1 Abdominal pain 1 Aphasia 1 Right hemiparesis 1 Seizures 1 Coma 1 Anaemia	2 Thrombocytopenia 1 High D-dimer 1 Low Hb 1 Positive for antibodies directed against (PF4) antibodies	Not performed [n = 1]	Extensive pulmonary artery and portal vein thrombosis [n = 2]	1 Mechanical thrombectomy 1 PRBCs 1 Decompressive craniectomy 2 Steroid 2 IVIG 1 Plasma exchange 1 Fondaparinux 1 Intubation	(NOS, high) 1 survived 1 died
Fanni et al. 2021 [98], Italy	Retrospective case report, single centre	58	0 (0)	1 White (Caucasian)	13	1 Not reported	Oxford Uni-AstraZeneca, dose 1 [n = 1]	New-onset [n = 1]	1 Abdominal pain 1 Diarrhea 1 Vomiting 1 Hepatic failure 1 Renal failure	1 Thrombocytopenia 1 Low fibrinogen 1 High D-dimer 1 High INR 1 High PT 1 High APTT 1 Low Hb	Voluminous fibrin thrombi in the branches of the portal vein [n = 1]	Portal vein thrombosis [n = 1] Splenic vein thrombosis [n = 1] Superior mesenteric vein thrombosis [n = 1]	Not reported [n = 1]	(NOS, moderate) 1 died
Graça et al. 2021 [96], Portugal	Retrospective case report, single centre	62	0 (0)	1 White (Caucasian)	1	1 Obesity 1 Asthma 1 Rhinitis	Oxford Uni-AstraZeneca, dose 1 [n = 1]	New-onset [n = 1]	1 Abdominal pain 1 Nausea 1 Vomiting 1 Fever 1 Epigastric tenderness 1 Iliac fossa tenderness	1 Low Hb 1 Thrombocytosis 1 High leucocytes 1 High CRP 1 Raised liver enzymes 1 Raised bilirubin	Not performed [n = 1]	Total occlusion at the hepatic and splenic arteries [n = 1]	1 Antibiotics 1 PRBCs 1 Heparin 1 Anticoagulant	(NOS, moderate) 1 survived
Graf et al. 2021 [3], Germany	Retrospective case report, single centre	29	1 (100)	1 White (Caucasian)	9	1 Not reported	Oxford Uni-AstraZeneca, dose 1 [n = 1]	New-onset [n = 1]	1 Headache 1 Abdominal pain 1 Abdominal cramps 1 Vomiting 1 Hematemesis 1 Multilocular thrombosis 1 Seizures 1 Intracranial hemorrhage 1 Aphasia 1 Apraxia	1 Thrombocytopenia 1 High D-dimer 1 Positive for antibodies directed against (PF4) antibodies	Not performed [n = 1]	Extensive thrombosis of the mesenteric and portal vein [n = 1]	1 IVIG 1 Argatroban	(NOS, high) 1 survived
Greenhall et al. 2021 [95], United Kingdom	Retrospective case reports, single centre	Median (IQR), 34 (21–63)	11 (85)	13 Whites (Caucasians)	Median (IQR), 10 (7–18)	13 DBD donors	Oxford Uni-AstraZeneca, dose 1 [n = 13]	New-onset [n = 13]	12 Intracranial haemorrhages 7 Cerebral venous sinus thrombosis 6 Extra-cranial thrombosis	4 Thrombocytopenia 5 High D-dimer	Not reported [n = 1]	Thrombosis of the portal veins [n = 2] Splenic vein thrombosis [n = 1]	Not reported [n = 13]	(NOS, high) 13 died
Kadam et al. 2022 [91], United Kingdom	Retrospective case reports, single centre	55	0 (0)	1 White (Caucasian)	14	1 Not reported	Oxford Uni-AstraZeneca, dose 1 [n = 1]	New-onset [n = 1]	1 Headache 1 Confusion 1 Abdominal pain 1 Reduced GCS 1 Reduced muscle power bilaterally 1 Dysphasia	1 Thrombocytopenia 1 Raised liver enzymes 1 Raised bilirubin 1 High PT 1 High INR 1 High D-dimer 1 Positive for antibodies directed against (PF4) antibodies	Not reported [n = 1]	Thrombosis of the portal and hepatic veins and multiple infarcts of the liver, left kidney and lingular segment of the partially imaged lungs [n = 1]	1 IVIG 1 Fresh frozen plasma 1 Anticoagulant 1 Exchange transfusion	(NOS, moderate) Outcome was not reported
Kulkarni et al. 2021 [59], India	Retrospective case report, single centre	46	1 (100)	1 Indian	7	1 Budd-Chiari syndrome 1 JAK2 positive myeloproliferative neoplasm 1 DIPS 1 Hypertension 1 Diabetes mellitus	Oxford Uni-AstraZeneca, dose 1 [n = 1]	New-onset [n = 1]	1 Abdominal pain	Not reported [n = 1]	Not performed [n = 1]	No flow in the DIPS stent [n = 1] Completely thrombosed portal vein, splenic vein, and DIPS stent [n = 1]	1 Thrombolysis 1 Venoplasty 1 Heparin 1 Dabigatran	(NOS, moderate) 1 survived
Lin et al. 2021 [58], Taiwan	Retrospective case report, single centre	42	0 (0)	1 Asian	5	1 Budd-Chiari syndrome	Oxford Uni-AstraZeneca, dose 1 [n = 1]	New-onset [n = 1]	1 Fever 1 Headache 1 Abdominal pain 1 Legs edema	1 Raised liver enzymes 1 Thrombocytopenia 1 High D-dimer 1 Positive for antibodies directed against (PF4) antibodies	Not performed [n = 1]	No flow in the right hepatic vein [n = 1] Thrombosis and occlusion in her right hepatic vein [n = 1]	1 IVIG 1 Anticoagulants 1 Steroid	(NOS, moderate) 1 survived
Major et al. 2022 [57], United States	Retrospective case report, single centre	50	1 (100)	1 White (Caucasian)	21	1 Obesity 1 Alcoholic cirrhosis	Johnson & Johnson COVID-19 vaccine, dose 1 [n = 1]	New-onset [n = 1]	1 Abdominal pain 1 Abdominal distension 1 Fatigue 1 Dark urine	1 Thrombocytopenia 1 Raised liver enzymes 1 Raised bilirubin 1 High INR 1 High D-dimer 1 High creatinine 1 Positive for antibodies directed against (PF4) antibodies	Not performed [n = 1]	Cirrhotic liver disease [n = 1] Complete thrombosis of the right portal vein [n = 1] Partial thrombus in the main portal vein [n = 1]	1 Argatroban 1 IVIG 1 Steroid 1 Bivalirudin 1 Rituximab 1 TIPS procedure 1 Plasma exchange 1 Fondaparinux	(NOS, moderate) 1 survived
Öcal et al. 2021 [4], Germany	Retrospective case reports, single centre	41	0 (0)	1 White (Caucasian)	11	1 No medical history	Oxford Uni-AstraZeneca, dose 1 [n = 1]	New-onset [n = 1]	1 Headache 1 Abdominal pain 1 Hypovolaemic shock	1 Thrombocytopenia 1 High D-dimer 1 Positive for antibodies directed against (PF4) antibodies	Not performed [n = 1]	Massive thrombosis of the entire portal venous system [n = 1] Splenomegaly [n = 1]	1 Anticoagulant 1 Analgesics 1 IVIG 1 Emergent laparotomy	(NOS, high) 1 survived
Premkumar et al. 2021 [56], India	Retrospective case-series, single centre	Median (IQR), 53 (48–53)	2 (66.7)	3 Indians	Not reported [n = 3]	1 NAFLD 1 Hepatitis C infection 1 Alcoholic cirrhosis 1 Diabetes mellitus	Oxford Uni-AstraZeneca, dose 1 [n = 2] Oxford Uni-AstraZeneca, dose 2 [n = 1]	New-onset [n = 3]	1 Pain 2 Ascites	Not reported [n = 3]	Not performed [n = 3]	Portal vein thrombosis [n = 2] Superior mesenteric vein thrombosis [n = 1]	1 Heparin 1 Dabigatran 1 Variceal eradication	(NOS, moderate) 2 survived 1 died
Ramdeny et al. 2021 [55], United Kingdom	Retrospective case report, single centre	54	1 (100)	1 Indian	21	1 Rare congenital limb malformation 1 Strong family history of a rare congenital limb deformity 1 Thrombophlebitis of the right leg	Oxford Uni-AstraZeneca, dose 1 [n = 1]	New-onset [n = 1]	1 Headache 1 Bruising 1 Unilateral right calf swelling	1 High D-dimer 1 Thrombocytopenia 1 Positive for antibodies directed against (PF4) antibodies	Not performed [n = 1]	Extensive cerebral venous sinus thrombosis [n = 1] Concurrent venous thrombosis in the portal vein [n = 1]	1 IVIG 1 Danaparoid 1 DOAC	(NOS, moderate) 1 survived
Repp et al. 2022 [116], United States	Retrospective case report, single centre	34	0 (0)	1 White (Caucasian)	5	1 Polycystic ovarian syndrome 1 Hypothyroidism 1 Smoking 1 Contraception 1 Family history of deep vein thrombosis	Moderna, dose 2 [n = 1]	New-onset [n = 1]	1 SOB 1 Abdominal pain 1 Dyspnea 1 Nausea 1 Diarrhea 1 Fever 1 Lightheadedness 1 Headache 1 Pruritus	Not reported [n = 1]	Portal vein thrombosis [n = 1]	Unremarkable [n = 1]	1 Analgesics 1 Ondansetron 1 IV fluids 1 Antacids 1 Rivaroxaban	(NOS, moderate) 1 survived
Schultz et al. 2021 [49], Norway	Retrospective case reports, single centre	32	1 (100)	1 White (Caucasian)	7	1 Asthma	Oxford Uni-AstraZeneca, dose 1 [n = 1]	New-onset [n = 1]	1 Back pain	1 Thrombocytopenia 1 High D-dimer 1 Positive for antibodies directed against (PF4) antibodies	Not performed [n = 1]	Thrombosis of several branches of the portal vein with occlusion of the left intrahepatic portal vein and left hepatic vein [n = 1] Thrombosis of the splenic vein, the azygos vein, and the hemiazygos vein [n = 1]	1 Platelet concentrate 1 IVIG 1 Steroid 1 Dalteparin 1 Warfarin	(NOS, high) 1 survived
Scully et al. 2021 [54], United Kingdom	Retrospective case-series, multicenter	Median (IQR), 54 (30–54)	1 (33.3)	3 Whites (Caucasians)	Mean (SD), 9.7 (3.5)	1 Deep vein thrombosis 1 Contraception	Oxford Uni-AstraZeneca, dose 1 [n = 3]	New-onset [n = 3]	Not reported [n = 3]	3 Thrombocytopenia 1 High PT 3 High APTT 3 Low fibrinogen 2 High D-dimer 3 Positive for antibodies directed against (PF4) antibodies	Thrombosis in many small vessels, especially vessels in the lungs and intestine, cerebral veins, and venous sinuses [n = 1] Extensive intracerebral hemorrhage [n = 1]	Cerebral venous thrombosis [n = 1] Portal vein thrombosis [n = 3] Pulmonary embolisms [n = 1] Middle cerebral artery infarcts [n = 1]	Not reported [n = 3]	(NOS, high) 1 survived 2 died
See et al. 2021 [53], United States	Retrospective case-series, multicenter	18–39 (n = 1) and ≥ 40 (n = 1)	0 (0)	2 Whites (Caucasians)	8 and 13	1 Obesity 1 Contraception	Johnson & Johnson COVID-19 vaccine, dose 1 [n = 2]	New-onset [n = 2]	2 Headache 2 Abdominal pain 1 Vomiting 1 Nausea 1 Myalgia 1 Chills 1 Fever 1 Back pain 1 Bruising 1 Malaise	2 Thrombocytopenia 2 High D-dimer 1 High APTT 1 High INR 2 Positive for antibodies directed against (PF4) antibodies	Not performed [n = 2]	Portal vein thrombosis [n = 2] Pulmonary embolus [n = 1] Intracerebral hemorrhage [n = 1] Retroperitoneal, intraperitoneal, and pelvic hemorrhage [n = 1] Thrombosis of the splenic vein [n = 1] Thrombosis of the right hepatic vein [n = 1] Thrombosis of the distal superior mesenteric vein [n = 1]	1 Aspirin 1 Paracetamol 1 Caffeine 1 Argatroban 1 IVIG	(NOS, high) 1 survived 1 died
Strobel et al. 2021 [5], Germany	Retrospective case report, single centre	29	1 (100)	1 White (Caucasian)	14	1 No medical history	Oxford Uni-AstraZeneca, dose 1 [n = 1]	New-onset [n = 1]	1 Abdominal pain 1 Headache 1 Skin petechia	1 High D-dimer 1 Thrombocytopenia 1 Positive for antibodies directed against (PF4) antibodies	Not performed [n = 1]	Thrombosis of the portal vein [n = 1] Thrombosis of the splenic vein [n = 1] Thrombosis of the superior mesenteric vein [n = 1]	1 Steroid 1 Argatroban 1 IVIG 1 Apixaban	(NOS, moderate) 1 survived
Thaler et al. 2021 [52], Austria	Retrospective case-series, multicenter	40 and 63	2 (100)	2 Whites (Caucasians)	7 and 17	2 No medical history	Oxford Uni-AstraZeneca, dose 1 [n = 2]	New-onset [n = 2]	1 Abdominal pain 1 Headache 1 Chills 1 Fever 1 Photophobia 1 Petechiae 1 Hematomas	2 High D-dimer 2 Thrombocytopenia 2 Positive for antibodies directed against (PF4) antibodies	Not performed [n = 2]	Thrombosis of the portal- and hepatic vein [n = 1] Thrombosis of the splenic-, and mesenteric vein [n = 1]	1 Rivaroxaban 1 IVIG 1 Fondaparinux 1 Steroid 1 Apixaban	(NOS, moderate) 2 survived
Tiwari et al. 2022 [31], India	Retrospective case report, single centre	24	0 (0)	1 Indian	18	1 Contraception 1 Menstrual irregularities	Oxford Uni-AstraZeneca, dose 1 [n = 1]	New-onset [n = 1]	1 Headache 1 Nausea 1 Vomiting 1 Seizures 1 Brain death 1 Absent brainstem reflexes 1 Positive apnea test	1 Thrombocytopenia 1 High D-dimer 1 High INR	Unremarkable [n = 1]	Venous sinus thrombosis [n = 1] Portal vein thrombosis [n = 1] Hemorrhagic transformation [n = 1]	1 Heparin 1 Digital subtraction angiography with thrombus extraction 1 IVIG 1 Intubation 1 MV	(NOS, moderate) 1 died
Tølbøll Sørensen et al. 2021 [51], Denmark	Retrospective case report, single centre	30	0 (0)	1 White (Caucasian)	8	1 Migraine 1 Contraception	Oxford Uni-AstraZeneca, dose 1 [n = 1]	New-onset [n = 2]	1 Headache 1 Malaise 1 Ecchymosis 1 Dizziness	1 Thrombocytopenia 1 Raised liver enzymes 1 High D-dimer 1 Positive for antibodies directed against (PF4) antibodies	Not performed [n = 1]	Portal vein thrombosis [n = 1]	1 Tinzaparin 1 Fibrinogen substitution 1 Fondaparinux 1 Rivaroxaban	(NOS, moderate) 1 survived
Umbrello et al. 2021 [76], Italy	Retrospective case report, single centre	36	0 (0)	1 White (Caucasian)	17	1 Fever 1 Asthenia 1 Osteoarticular pain 1 Melena	Oxford Uni-AstraZeneca, dose 1 [n = 1]	New-onset [n = 1]	1 Abdominal pain 1 Low blood pressure 1 High heart rate	1 Thrombocytopenia 1 Positive for antibodies directed against (PF4) antibodies 1 Low Hb	Not performed [n = 1]	Complete thrombosis of spleno-mesenteric-portal axis [n = 1]	1 Heparin 1 Thrombus aspiration 1 Porto-systemic shunt 1 IV rtPA thrombolysis 1 Argatroban 1 IVIG 1 PRBCs 1 Epinephrine 1 Apixaban	(NOS, moderate) 1 survived
Uzun et al. 2022 [30], Germany	Retrospective case report, single centre	50	0 (0)	1 White (Caucasian)	12	1 Not reported	Johnson & Johnson COVID-19 vaccine, dose not reported [n = 1]	New-onset [n = 1]	1 Vaccine-induced immune thrombotic thrombocytopenia 1 Thrombocytopenia and thrombosis of the cerebral arteries and venous sinuses 1 Brain death	1 Thrombocytopenia 1 High D-dimer 1 Positive for antibodies directed against (PF4) antibodies 1 High creatinine	Hemangioma and a segment with an arterial thrombosis [n = 1] Intraluminal blood clot was detected in the liver after organ procurement [n = 1]	Occlusion of the middle cerebral artery [n = 1] Sinus vein thrombosis of the superior sagittal sinus and transverse sinus [n = 1]	1 IVIG 1 Argatroban	(NOS, moderate) 1 died
*Raised liver enzymes*														
Alkindi et al. 2021 [114], Oman	Retrospective case reports, single centre	Median (IQR), 29 (28–29)	3 (100)	3 Arabs	Mean (SD), 5 (1)	1 Avascular necrosis of shoulders 2 Splenectomy 2 Cholecystectomy 2 Acute chest syndrome 1 Tuberculosis of the spine	Oxford Uni-AstraZeneca, dose 1 [n = 3]	New-onset [n = 3]	1 Shoulder pain 2 Back pain 1 Fever 1 Chest pain 1 Tachypnea 1 Tachycardia 2 Low saturation	3 Raised liver enzymes 3 High CRP 1 Raised bilirubin 2 Low Hb 2 Thrombocytopenia 1 Hyponatremia 1 High D-dimer	Not performed [n = 3]	Right-sided infiltrates [n = 1] Pleural effusion [n = 1]	1 Analgesics 2 Antibiotics 1 PRBCs 1 Heparin 1 Exchange transfusion 1 CPR 1 Oxygen supplementation 1 Thrombolysis	(NOS, low) 2 survived 1 died
Alrashdi et al. 2022 [39], Saudi Arabia	Retrospective case report, single centre	22	0 (0)	1 Arab	7	1 No medical history	Pfizer-BioNTech, dose 1 [n = 1]	New-onset [n = 1]	1 Abdominal pain 1 Nausea 1 Vomiting 1 Maculopapular rash over extremities 1 Systemic lupus erythematosus	1 Leukopenia 1 Lymphopenia 1 Raised white blood cells 1 Hemolytic anemia 1 Low Hb 1 High reticulocyte count 1 Thrombocytopenia 1 High LDH 1 Raised liver enzymes 1 Raised bilirubin 1 High pancreatic enzymes 1 High ESR 1 Hypocomplementemia 1 Positive ANAs 1 Positive anti-double strand deoxyribonucleic acid	Not performed [n = 1]	Autoimmune pancreatitis [n = 1]	1 Steroid 1 Azathioprine 1 HCQ	(NOS, moderate) 1 survived
Brown et al. 2022 [38], United States	Retrospective case report, single centre	58	1 (100)	1 White (Caucasian)	7	1 Obesity 1 Hypertension	Pfizer-BioNTech, dose 2 [n = 1]	New-onset [n = 1]	1 Headache 1 Nausea 1 Myalgias 1 Fever 1 Chills 1 Sweats 1 Diarrhea 1 Anxiety 1 Encephalopathic 1 Rash 1 Splenomegaly 1 Hypotension 1 NSTEMI (Type 2) 1 Acute interstitial nephritis 1 Acute tubular necrosis 1 Multisystem inflammation syndrome	1 High CRP 1 High ferritin 1 Acute kidney injury 1 Raised liver enzymes 1 Raised bilirubin 1 High hs-cTnT 1 Pancytopenia 1 Low Hb	Not performed [n = 1]	Unremarkable [n = 1]	1 Intubation 1 Steroid	(NOS, moderate) 1 survived
Chai et al. 2022 [9], Denmark	Retrospective case report, single centre	17	1 (100)	1 White (Caucasian)	5	1 No medical history	Pfizer-BioNTech, dose 2 [n = 1]	New-onset [n = 1]	1 Fever 1 Vomiting 1 Myalgia 1 Chest pain 1 Fatigue 1 Multisystem inflammation syndrome 1 Myocarditis	1 Raised liver enzymes	Not performed [n = 1]	Myocarditis [n = 1]	1 Norepinephrine 1 Oxygen supplementation 1 Steroids 1 IVIG 1 Antibiotics	(NOS, moderate) 1 survived
Cirillo et al. 2022 [46], Italy	Retrospective case report, single centre	68	1 (100)	1 White (Caucasian)	9	1 No medical history	Oxford Uni-AstraZeneca, dose 1 [n = 1]	New-onset [n = 1]	1 Hypoglycemia 1 Malaise 1 Dyspnea 1 Abdominal pain 1 Difficulty with walking 1 Untreatable hypotensive shock 1 Contraction of diuresis 1 Clouding of the sensory 1 Weakness in the four limbs 1 Atrial fibrillation 1 Rhabdomyolysis 1 Kidney injury 1 Respiratory failure 1 Bone marrow failure 1 Multi-organ failure	1 Multi-lineage cytopenia 1 High procalcitonin 1 Increase of myoglobin 1 Raised liver enzymes 1 High D-dimer 1 High LDH 1 High creatine kinase 1 High creatinine 1 High blood urea nitrogen 1 Hyperkalemia 1 Hypocalcemia 1 Lymphopenia 1 High lactic acid 1 Isolation of *Pseudomonas aeruginosa* from bronchial aspirate	Fiber necrosis with phagocytosis and influx of histiocytes, associated with a significant increase of the vascular component [n = 1]	Severe interstitial pneumopathy [n = 1] Severe bilateral pleural effusion [n = 1]	1 Steroid 1 Anakinra 1 Eculizumab 1 Beta blockers 1 Hemodialysis 1 Intubation 1 Tracheostomy 1 Meropenem 1 Amphotericin B 1 Tigecycline 1 Fosfomycin 1 Cotrimoxazole	(NOS, high) 1 died
Fritzen et al. 2022 [36], Brazil	Retrospective case report, single centre	60	0 (0)	1 Hispanic	11	1 Chronic liver disease 1 Portal hypertension 1 Polycythemia vera 1 Hypothyroidism 1 Diabetes mellitus	Oxford Uni-AstraZeneca, dose 2 [n = 1]	New-onset [n = 1]	1 Painful purpuric lesions 1 Palpable papules 1 Leukocytoclastic vasculitis	1 Raised liver enzymes 1 High CRP 1 High leukocytes 1 High APTT 1 High INR 1 High LDH	The histological picture was compatible with leukocytoclastic vasculitis [n = 1]	Not performed [n = 1]	1 Steroid	(NOS, moderate) 1 survived
Gadi et al. 2021 [27], United States	Retrospective case report, single centre	41	0 (0)	1 White (Caucasian)	7	1 Central retinal vein occlusion 1 Hypertension	Pfizer-BioNTech, dose 1 [n = 1]	New-onset [n = 1]	1 Autoimmune hemolytic anemia 1 Fatigue 1 Dark urine 1 Dyspnea 1 Anxiety	1 Thrombocytopenia 1 Low Hb 1 High reticulocyte count 1 Raised white blood cells 1 Raised liver enzymes 1 Raised bilirubin 1 High LDH 1 Positive direct antiglobulin test for IgG	Not performed [n = 1]	Not performed [n = 1]	1 PRBCs 1 Steroid 1 Rituximab 1 Mycophenolate mofetil 1 IVIG	(NOS, moderate) 1 survived
Gaignard et al. 2021 [26], Switzerland	Retrospective case report, single centre	77	1 (100)	1 White (Caucasian)	5	1 No medical history	Moderna, dose 1 [n = 1]	New-onset [n = 1]	1 Weakness 1 Fatigue 1 SOB 1 Autoimmune hemolytic anemia	1 High reticulocyte count 1 High leukocytes 1 Raised liver enzymes 1 Raised bilirubin 1 High LDH 1 Positive tests for indirect antiglobulin, IgG, complement component 3 and direct antiglobulin	Not performed [n = 1]	Discrete inhomogeneous liver parenchyma [n = 1]	1 Steroid	(NOS, moderate) 1 survived
Hines et al. 2021 [94], United States	Retrospective case report, single centre	26	0 (0)	1 White (Caucasian)	14	1 Irregular menses 1 Contraception	Moderna, dose 1 [n = 1]	New-onset [n = 1]	1 Rash 1 Bruising 1 Urticaria	1 Thrombocytopenia 1 Raised liver enzymes	Unremarkable [n = 1]	Unremarkable [n = 1]	1 Steroid 1 IVIG 1 N-acetylcysteine	(NOS, moderate) 1 survived
Jawed et al. 2021 [93], United States	Retrospective case report, single centre	47	0 (0)	1 White (Caucasian)	18	1 Hashimoto’s thyroiditis 1 Anaemia 1 Lymphadenopathy 1 ITP	Pfizer-BioNTech, dose 1 [n = 1]	Relapsed [n = 1]	1 Easy bruising 1 Gum bleeding 1 Epistaxis 1 Ecchymosis 1 Petechiae	1 Thrombocytopenia 1 Raised liver enzymes 1 High PT 1 High INR 1 High LDH 1 High reticulocyte count 1 Positive ANAs 1 Positive anti–Sjögren syndrome antigen A	Not performed [n = 1]	Unremarkable [n = 1]	1 Steroid 1 IVIG	(NOS, moderate) 1 survived
Khajavirad et al. 2022 [33], Iran	Retrospective case report, single centre	70	0 (0)	1 Persian	1	1 Diabetes mellitus 1 Hypertension 1 Coronary artery disease 1 Percutaneous coronary intervention	Oxford Uni-AstraZeneca, dose 1 [n = 1]	New-onset [n = 1]	1 Headache 1 Generalized tonic–clonic seizure 1 Lethargy 1 Anaemia	1 Raised liver enzymes 1 High leukocytes 1 High LDH 1 High creatinine 1 High CRP 1 High ESR 1 Thrombocytopenia 1 High D-dimer 1 Low Hb 1 Positive for antibodies directed against (PF4) antibodies	Not performed [n = 1]	Acute infarction in left occipital lobe [n = 1]	1 Paracetamol 1 IVIG 1 Steroid 1 Rivaroxaban 1 Sodium valproate 1 Levetiracetam 1 Anticoagulants	(NOS, moderate) 1 survived
Kishimoto et al. 2022 [70], Japan	Retrospective case report, single centre	46	1 (100)	1 Asian	10	1 Hyperlipidemia 1 Alcohol-associated liver disease	Moderna, dose 1 [n = 1]	New-onset [n = 1]	1 Fever 1 Odynophagia 1 Bilateral anterior neck pain 1 Enlarged thyroid 1 Thyroid tenderness 1 Subacute thyroiditis	1 Low TSH 1 Elevated FT-3 1 Elevated F-T4 1 High CRP 1 Raised liver enzymes	Not performed [n = 1]	Fatty liver [n = 1] Gallbladder polyps [n = 1]	1 Steroid	(NOS, moderate) 1 survived
Kyungu et al. 2022 [121], Democratic Republic of the Congo	Retrospective case report, single centre	29	1 (100)	1 Black	2	1 No medical history	Johnson & Johnson COVID-19 vaccine, dose 1 [n = 1]	New-onset [n = 1]	1 Headache 1 Nausea 1 Fever 1 Abdominal pain 1 Dark urine 1 Acute hepatitis 1 Acute cholecystitis	1 High CRP 1 Raised liver enzymes 1 Thrombocytopenia 1 Leukopenia	Not performed [n = 1]	Thickened gallbladder wall without evidence of gallstones [n = 1] Positive Murphy´s sonographic sign [n = 1]	1 IV fluids 1 Analgesics 1 Antibiotics 1 Rabeprazole	(NOS, moderate) 1 survived
Malayala et al. 2021 [89], United States	Retrospective case report, single centre	60	1 (100)	1 Black	2	1 Hepatitis C infection 1 Chronic kidney disease 1 Hypertension 1 Congestive heart failure 1 Smoking	Moderna, dose 1 [n = 1]	Relapsed [n = 1]	1 Generalized weakness 1 SOB 1 Leg edema 1 Nausea 1 Vomiting 1 Abdominal pain 1 Chest pain 1 Rash	1 High creatinine 1 Thrombocytopenia 1 Raised liver enzymes 1 Raised bilirubin 1 High INR 1 High ferritin 1 High LDH 1 high CRP	Not performed [n = 1]	Liver cirrhosis [n = 1]	1 Antihypertensives 1 Diuretics	(NOS, moderate) Outcome is unknown
Mücke et al. 2021 [25], Germany	Retrospective case report, single centre	76	1 (100)	1 White (Caucasian)	12	1 Compensated alcoholic liver cirrhosis 1 Heart failure 1 Gastrectomy 1 Gastroesophageal junction cancer 1 Prostatectomy 1 Prostate cancer 1 Indwelling suprapubic catheter 1 Lesions on hands and feet	Pfizer-BioNTech, dose 2 [n = 1]	New-onset [n = 1]	1 Pruritus 1 Swelling 1 Limb swelling 1 Purpuric rash 1 Palpable maculae on both hands, legs and thighs 1 Melaena 1 Diarrhoea 1 Myalgia 1 Fever 1 Hoarseness 1 Fatigue 1 Vaccine-induced cutaneous and gastrointestinal immune complex vasculitis	1 High ESR 1 High interleukin-6 levels 1 High CRP 1 Micro-erythruria 1 Leukocyturia 1 Positive fecal occult test 1 High calprotectin 1 Raised liver enzymes	Not performed [n = 1]	Unremarkable [n = 1]	1 Steroid	(NOS, moderate) 1 survived
O'Connor et al. 2022 [32], Ireland	Retrospective case report, single centre	45	0 (0)	1 White (Caucasian)	49	1 No medical history	Oxford Uni-AstraZeneca, dose 1 [n = 1]	New-onset [n = 1]	1 Rash 1 Chills 1 Malaise 1 Conjunctivitis 1 Generalized erythema 1 Sore throat 1 Hoarseness 1 Erythema of the eyelids 1 Edema of the lips 1 Papules and plaques on the face, trunk, and extremities 1 Pustules on the upper lip 1 Edema of the arms and legs 1 Conjunctivitis 1 Erythema of the pharynx 1 Cervical lymphadenopathy	1 Elevated eosinophil 1 High CRP 1 Raised liver enzymes	Drug reaction with eosinophilia and systemic symptoms syndrome [n = 1]	Serositis [n = 1] Mild fluid in the pleural and peritoneal cavities [n = 1]	1 Levocetirizine 1 Fexofenadine 1 Steroid	(NOS, moderate) 1 survived
Sauret et al. 2022 [23], France	Retrospective case report, single centre	70	1 (100)	1 White (Caucasian)	A few days	1 Not reported	Oxford Uni-AstraZeneca, dose 1 [n = 1]	New-onset [n = 1]	1 Headache 1 Hyperesthesia of the scalp	1 Raised liver enzymes 1 High CRP 1 High APTT	Gant cell arteritis [n = 1]	Unremarkable [n = 1]	1 Steroid	(NOS, moderate) 1 survived
Sharabi et al. 2021 [79], Israel	Retrospective case report, single centre	56	0 (0)	1 Jew	7	1 Not reported	Pfizer-BioNTech, dose 2 [n = 1]	New-onset [n = 1]	1 SOB 1 Chest pain 1 Weakness 1 Fever 1 Sore throat 1 Pain 1 Swelling of joints, knees and ankles 1 Tachycardia 1 Dyspnea 1 Rash	1 Raised liver enzymes 1 Raised bilirubin 1 High leukocytes 1 Hypoalbuminemia 1 High hs-cTnT 1 High ferritin	Not performed [n = 1]	Unremarkable [n = 1]	1 Steroid	(NOS, moderate) 1 survived
Sung et al. 2022 [40], Republic of Korea	Retrospective case report, single centre	34	0 (0)	1 Asian	42	1 No medical history	Pfizer-BioNTech, dose 1 [n = 1]	New-onset [n = 1]	1 Increased abdominal circumference 1 Pitting oedema of the lower extremities 1 Splenomegaly 1 Ascites 1 Budd-Chiari syndrome	1 Raised liver enzymes 1 High D-dimer	Dilated sinusoids with extensive perisinusoidal hepatocyte dropout [n = 1]	Collapsed hepatic veins and decreased portal vein flow [n = 1] Pulmonary thromboembolism [n = 1]	1 IVIG 1 Anticoagulants	(NOS, moderate) 1 survived
Tan et al. 2021 [77], United Kingdom	Retrospective case report, single centre	34	1 (100)	1 White (Caucasian)	1	1 CPT II deficiency	Oxford Uni-AstraZeneca, dose 1 [n = 1]	New-onset [n = 1]	1 Fever 1 Vomiting 1 SOB 1 Hematuria 1 Myalgia 1 Muscle weakness	1 Raised liver enzymes 1 Raised white blood cells 1 High creatine kinase 1 Low adjusted calcium	Not performed [n = 1]	Unremarkable [n = 1]	1 IV dextrose 1 Carbohydrate-rich diet 1 Paracetamol	(NOS, moderate) 1 survived
Waqar et al. 2021 [83], United States	Retrospective case report, single centre	69	0 (0)	1 White (Caucasian)	7	1 Hypertension 1 Chronic kidney disease 1 AIDS 1 Chronic hepatitis B 1 DVT	Pfizer-BioNTech, dose 2 [n = 1]	New-onset [n = 1]	1 Fatigue 1 SOB 1 Fever 1 Chills 1 Night sweats 1 Weight loss 1 Headaches 1 Vision changes 1 Cough 1 Sputum chest pain 1 Abdominal pain 1 Rash 1 Bleeding 1 Bruising 1 Oedema 1 Focal weakness 1 Changes in bowel or urinary habits	1 Anaemia 1 Thrombocytopenia 1 Raised liver enzymes 1 Raised bilirubin 1 High reticulocyte count 1 High LDH	Not performed [n = 1]	Unremarkable [n = 1]	1 Exchange transfusion 1 Steroid 1 Plasmapheresis 1 Rituximab	(NOS, moderate) 1 survived
Watanabe et al. 2022 [10], Japan	Retrospective case report, single centre	51	0 (0)	1 Asian	2	1 No medical history	Pfizer-BioNTech, dose 2 [n = 1]	New-onset [n = 1]	1 Fever 1 Genital bleeding 1 Petechia 1 DIC 1 Macrophage activation syndrome 1 Plasmacytoid dendritic cells	1 Thrombocytopenia 1 Reduced white blood cells 1 Pancytopenia 1 Raised liver enzymes 1 Raised bilirubin 1 High LDH 1 High CRP 1 High blood urea nitrogen 1 High creatinine 1 High D-dimer 1 High ferritin	Plasmacytoid dendritic cells [n = 1]	Not performed [n = 1]	1 Steroid 1 IVIG	(NOS, moderate) 1 survived
Wu et al. 2022 [28], United States	Retrospective case report, single centre	77	0 (0)	1 Hispanic	5	1 No medical history	Pfizer-BioNTech, dose 1 [n = 1]	New-onset [n = 1]	1 Muscle aches 1 Weakness 1 Fever 1 Pruritic and painful eruption on the right and left arms, chest and neck 1 Violaceous, poikilodermatous scaly plaques 1 Multiple vesicles and erythematous papules and patches on both thighs	1 High creatinine 1 Raised liver enzymes 1 Elevated anti-transcription intermediary factor 1γ antibody levels	Dermatomyositis [n = 1]	Unremarkable [n = 1]	1 IVIG 1 Steroid 1 Mycophenolate mofetil	(NOS, moderate) 1 survived
Yocum et al. 2021 [11], United States	Retrospective case reports, single centre	62	0 (0)	1 White (Caucasian)	37	1 Hypertension 1 Hyperlipidemia 1 Hypothyroidism 1 Gastroesophageal reflux disease	Johnson & Johnson COVID-19 vaccine, dose 1 [n = 1]	New-onset [n = 1]	1 Altered mental status 1 Petechiae 1 Vomiting 1 Acute kidney injury	1 Raised liver enzymes 1 Raised bilirubin 1 Raised white blood cells 1 High CRP 1 Thrombocytopenia 1 Low fibrinogen 1 High creatinine 1 High BUN 1 High LDH 1 Low Hb 1 High hs-cTnT	Not performed [n = 1]	Unremarkable [n = 1]	1 Intubation 1 Steroid 1 Hemodialysis 1 PRBCs 1 Plasma exchange	(NOS, moderate) Outcome was not reported
*Splanchnic vein thrombosis*														
Greinacher et al. 2021 [60], Germany and Austria	Retrospective case-series, multicenter	Median (IQR), 36 (22–49)	2 (18.2)	11 Whites (Caucasians)	Mean (SD), 9.3 (3.3)	8 No medical history 1 von Willebrand disease 1 Anticardiolipin antibodies 1 Factor V Leiden	Oxford Uni-AstraZeneca, dose 1 [n = 1] Oxford Uni-AstraZeneca, dose not reported [n = 10]	New-onset [n = 11]	1 Fatigue 1 Myalgia 1 Headache 1 Chills 1 Fever 1 Nausea 1 Epigastric discomfort 1 Tachycardia 1 Gastrointestinal haemorrhage 1 Ascites	1 Raised liver enzymes 11 Thrombocytopenia 7 High D-dimer 1 High LDH 1 High CRP 1 Low Hb 5 High INR 5 High APTT 4 Low fibrinogen 11 Positive for antibodies directed against (PF4) antibodies	Cerebral venous thrombosis [n = 1]	Cerebral venous thrombosis [n = 9] Intracranial hemorrhage [n = 1] Splanchnic-vein thrombosis [n = 3] Pulmonary embolisms [n = 3] DIC [n = 5] Other thromboses [n = 4]	1 Platelet concentrate 1 Antibiotics 1 Analgesics 5 Heparin 1 PRBCs 1 Prothrombin complex concentrates 1 Recombinant factor VIIa 1 Apixaban	(NOS, high) 5 survived 6 died
Muir et al. 2021 [50], United States	Retrospective case report, single centre	48	0 (0)	1 White (Caucasian)	14	1 No medical history	Johnson & Johnson COVID-19 vaccine, dose 1 [n = 1]	New-onset [n = 1]	1 Malaise 1 Abdominal pain 1 Anaemia 1 Headache	1 Thrombocytopenia 1 DIC 1 Low fibrinogen 1 High APTT 1 High D-dimer 1 Positive for antibodies directed against (PF4) antibodies	Not performed [n = 1]	Extensive splanchnic-vein thrombosis [n = 1] Haemorrhagic stroke of the brain [n = 1] New thrombus involving the right hepatic and splenic veins [n = 1]	1 Heparin 1 Argatroban 1 IVIG	(NOS, high) 1 survived
Tiede et al. 2021 [48], Germany	Retrospective case-series, single centre	61	0 (0)	1 White (Caucasian)	6	1 No medical history	Oxford Uni-AstraZeneca, dose 1 [n = 1]	New-onset [n = 1]	1 Fatigue	1 Thrombocytopenia 1 High D-dimer 1 Positive for antibodies directed against (PF4) antibodies	Not performed [n = 1]	Extensive splanchnic vein thrombosis [n = 1]	1 Argatroban 1 IVIG 1 Alteplase 1 Eculizumab	(NOS, moderate) 1 survived
van Dijk et al. 2022 [47], The Netherlands	Retrospective case report, single centre	61	0 (0)	1 White (Caucasian)	14	1 No medical history	Oxford Uni-AstraZeneca, dose 1 [n = 1]	New-onset [n = 1]	1 Abdominal pain 1 Nausea	1 Thrombocytopenia 1 High D-dimer 1 High CRP	Not performed [n = 1]	Extensive splanchnic vein thrombosis in the superior mesenteric vein, splenic vein and portal vein [n = 1]	1 Paracetamol 1 Rivaroxaban 1 IVIG	(NOS, moderate) 1 survived

*ACRL* Acute cellular rejection of the liver; *AIDS* acquired immunodeficiency syndrome; *AIH* autoimmune hepatitis; *AMAs* anti-mitochondrial antibodies; *ANAs* antinuclear antibodies; *anti-SLA* anti-soluble liver antigen; *APTT* activated partial thromboplastin time; *ARDS* acute respiratory distress syndrome; *ASMAs* anti-smooth muscle antibodies; *ATA* anti-thyroglobulin antibodies; *COVID-19* coronavirus disease 2019; *CPR* cardiopulmonary resuscitation; *CPT II* carnitine palmitoyltransferase II deficiency; *CRP* C-reactive protein; *CT* computed tomography; *DBD* donation after brain death; *DIC* disseminated intravascular coagulation; *DIPS* direct intrahepatic portosystemic shunt; *ds-DNA* double-stranded DNA antibodies; *DOAC* direct oral anticoagulant; *DVT* deep vein thrombosis; *ERCP* endoscopic retrograde cholangiopancreatography; *ESR* erythrocyte sedimentation rate; *F-T4* free thyroxine; *FFP* fresh-frozen plasma; *FT-3* free triiodothyronine; *GCS* Glasgow Coma Scale; *Hb* hemoglobin; *HCC* hepatocellular carcinoma; *HCQ* hydroxychloroquine; *hs-cTnT* high-sensitivity cardiac troponin test; *IgG* immunoglobulin G; *IgM* immunoglobulin M; *INR* international normalized ratio; *IV* intravenous; *IVIG* IV immunoglobulin; *ITP* immune thrombocytopenia; *LC-1* liver cytosolic antigen type 1; *LDH* lactate dehydrogenase; *MV* mechanical ventilation; *MRI* magnetic resonance imaging; *NAFLD* nonalcoholic fatty liver disease; *NSTEMI* non-ST-elevation myocardial infarction; *NOS* Newcastle Ottawa Scale; *PF4* platelet factor 4; *PRBCs* Transfusions of Packed Red Blood Cells; *PT* prothrombin time; *SD* standard deviation; *SIADH* syndrome of inappropriate antidiuretic hormone secretion; *SOB* shortness of breath; *TIPS* transjugular intrahepatic portosystemic shunt; *TSH* thyroid stimulating hormone

^a^Data are presented as median (25–75th percentiles)

^b^Patients with black ethnicity include African-American, Black African, African and Afro-Caribbean patients

^c^Biopsy findings are reported based on each institution’s written report. Biopsies were not independently reviewed

### Autoimmune hepatitis

Autoimmune hepatitis (AIH) was the first most-common liver disease reported following COVID-19 vaccination [eighty-three new onset cases [6–8, 37, 41, 68, 84, 85, 87, 97, 99, 101–108, 110, 112, 115, 117–120, 123, 124, 126, 127] and four previously known cases [43, 80, 86, 104]; and in fifty-one cases event if new-onset or relapsed was not reported [42]] (see Table 1). Most common clinical presentations in these AIH cases were fatigue (n = 75) [99, 102–104, 112, 118, 119, 124, 126, 127], jaundice (n = 68), [6–8, 37, 42, 68, 84, 85, 97, 99, 102, 104–108, 110, 112, 115, 117, 118, 123, 126, 127], nausea (n = 60) [68, 108, 112, 123, 126, 127], abdominal pain (n = 25) [7, 37, 68, 105, 126], pruritus (n = 10) [6, 37, 99, 101, 105, 110, 117, 127], itching (n = 10) [126], dark urine (n = 10) [6, 7, 68, 84, 103, 104, 106, 108, 110, 123], hepatomegaly (n = 6) [6, 7, 85, 102, 103, 123], fever (n = 5) [84, 104, 117, 123], malaise (n = 4) [84, 85, 97, 112], anorexia (n = 4) [8, 102, 104, 112], and yellow eyes (n = 4) [8, 103, 112, 118]. Four of the AIH cases were asymptomatic [43, 80, 86, 87]. The median interquartile range (IQR) age of this group was 59 [41 to 72], with an increased female predominance in AIH patients diagnosed after COVID-19 vaccination in most of the studies [n = 90, 65.2%] [6–8, 43, 68, 80, 84, 86, 87, 97, 99, 103, 105–108, 110, 112, 115, 118–120, 123, 124, 126], and majority of the patients belonged to White (Caucasian) (n = 34, 24.6%) [6, 7, 41–43, 68, 80, 85–87, 97, 99, 102, 103, 105–108, 112, 120, 127] and Asian (n = 13, 9.4%) [8, 84, 110, 115, 117–119, 123, 124] ethnicity. The median (IQR) time between the COVID-19 vaccination and time of presentation was 14 (7–20) days. Seventy-seven, twenty-nine, and twenty-nine of these one hundred-thirty eight cases were reported following Pfizer-BioNTech (eight after the first dose, eight after the second dose and three after the third dose) [6, 41, 43, 68, 84, 87, 99, 105, 106, 112, 115, 119, 120, 123, 124, 127], Moderna (nine after the first dose and three after the second dose) [7, 8, 80, 85, 97, 99, 102, 103, 107, 108, 117, 126], and Oxford Uni-AstraZeneca (three after the first dose, two after the second dose and one after the third dose) [37, 86, 99, 101, 115, 126] vaccination; respectively. Ten AIH patients had a history of thyroid gland disorders [Hashimoto’s thyroiditis (n = 6) [42, 103, 106, 112] and hypothyroidism (n = 4) [68, 86, 104, 127]] and seven patients had no medical history (n = 7, 5.1%) [85, 97, 110, 115, 117, 119, 123], however, some of the patients had a past medical history of hypertension (n = 17, 12.3%) [6, 101, 112, 118, 126], diabetes mellitus (n = 15, 10.9%) [102, 104, 126], hyperlipidaemia (n = 6, 4.3%) [8, 84, 105, 106, 115, 118], and rheumatoid arthritis (n = 2, 1.4%) [42]. Some of those AIH cases presented with a previous known history of hepatic pathologies [undetermined pre-existing liver disease (n = 12, 8.7%) [126], nonalcoholic fatty liver disease (n = 7, 5.1%) [126], primary biliary cholangitis (n = 5, 3.6%) [41, 80, 84, 115, 126], hepatitis C infection (n = 2, 1.4%) [124, 126], liver transplant recipient (n = 2, 1.4%) [43, 126], hepatitis B infection (n = 1, 0.7%) [120], autoimmune hepatitis (n = 1, 0.7%) [43], jaundice (n = 1, 0.7%) [104], liver cirrhosis (n = 1, 0.7%) [41], or hypertransaminasemia (n = 1, 0.7%) [86]]. Radiological imaging was unremarkable for a high number of the AIH cases (n = 22, 15.9%) [6, 8, 43, 68, 80, 85–87, 97, 99, 102, 104, 105, 108, 110, 115, 117, 119, 120, 124] or not reported (n = 100, 72.5%) [41, 42, 126], nevertheless, liver biopsy revealed histopathological findings consistent with AIH in all cases except for one patient [42]. Patients who suffered AIH post-COVID-19 vaccination were more likely to have positive antinuclear antibodies (n = 92) [6–8, 37, 41, 42, 80, 84–87, 97, 99, 101–104, 106, 108, 110, 112, 115, 117–119, 123, 124, 126], elevated immunoglobulin G (n = 89) [7, 8, 37, 41, 68, 80, 84–87, 97, 101–108, 110, 112, 115, 117–120, 123, 124, 126], raised liver enzymes (n = 55) [6–8, 37, 41–43, 68, 80, 84–87, 97, 99, 101–108, 110, 112, 115, 117–120, 123, 124, 126, 127], raised bilirubin (n = 41) [6–8, 37, 41, 42, 68, 80, 84, 85, 97, 99, 101–108, 110, 112, 115, 117, 118, 123, 124], positive anti-smooth muscle antibodies (n = 24) [8, 37, 42, 97, 103, 107, 108, 112, 118, 126], or high international normalized ratio (n = 6) [80, 84, 99, 104]. As expected, most prescribed pharmacotherapy agents in these AIH cases were steroids (n = 82) [6–8, 37, 41, 43, 68, 80, 84–87, 97, 99, 101–108, 110, 112, 115, 118, 120, 123, 124, 126, 127] and azathioprine (n = 20) [37, 41, 43, 68, 80, 86, 97, 103, 110, 112, 118, 126], however, pharmacotherapy was not reported in a high number of these AIH patients (n = 12, 8.7%) [42]. Clinical outcomes of the AIH patients with mortality were documented in 3 (2.2%) [99, 104, 115], while 123 (89.1%) of the AIH cases recovered [6–8, 37, 41, 43, 68, 80, 84–87, 97, 99, 101–108, 110, 112, 115, 117–120, 123, 124, 126, 127] and final treatment outcome was not reported in many AIH patients (n = 12, 29.3%) [42].

### Portal vein thrombosis

Portal vein thrombosis (PVT) was the second most common liver pathology reported following COVID-19 vaccination (fifty-two new-onset cases), with extra-cranial thrombosis (n = 21) [3, 5, 52–54, 56, 59, 61, 63–67, 76, 91, 95, 98, 111], headache (n = 20) [3–5, 31, 51–53, 55, 58, 64–67, 91, 111, 116], intracranial hemorrhage (n = 17) [3, 31, 53, 54, 95, 111], abdominal pain (n = 16) [3–5, 52, 53, 57–59, 61, 65, 76, 91, 96, 98, 116], cerebral venous sinus thrombosis (n = 13) [30, 31, 54, 55, 66, 95, 109], vomiting (n = 8) [3, 31, 53, 64, 67, 96, 98], fever (n = 8) [52, 53, 58, 64, 67, 96, 116], nausea (n = 6) [31, 53, 66, 96, 116] and seizures (n = 5) [3, 31, 61, 67, 111] as the common clinical presentations in these cases (see Table 1). The median interquartile range (IQR) age of this group was 47.5 (32.5 to 55) years, with an increased female predominance in PVT patients diagnosed after COVID-19 vaccination in most of the studies [n = 28, 53.8%] [4, 30, 31, 51, 53, 58, 61, 62, 64, 65, 67, 76, 91, 96, 98, 109, 111, 116], and majority of the patients belonged to White (Caucasian) (n = 44, 84.6%) [3–5, 30, 49, 51–54, 57, 61–66, 76, 91, 95, 96, 98, 109, 111, 116] and Indian (n = 6, 11.8%) [31, 55, 56, 59] ethnicity. The median (IQR) time between the COVID-19 vaccination and time of presentation was 10 (7–13) days. Forty-five of these fifty-one cases (forty-four after the first dose and one after the second dose) were reported following Oxford Uni-AstraZeneca vaccination [3–5, 31, 49, 51, 52, 54–56, 58, 59, 61, 62, 64, 65, 67, 76, 91, 95, 96, 98, 109, 111]. The remaining six PVT cases were reported after Johnson & Johnson COVID-19 vaccination [30, 53, 57, 63, 66]. Fourteen PVT patients were donors after brain death (n = 14, 27.4%) [95, 109] and seven patients had no medical history (n = 7, 13.7%) [4, 5, 52, 64, 66], however, some of the patients had a past drug history of regular intake of oral contraceptive pills (n = 6, 11.5%) [31, 51, 53, 54, 64, 111, 116]. Few PVT patients had pre-existing diabetes mellitus (n = 3) [56, 59, 67], migraine (n = 3) [51, 65, 111], thyroid gland disorders [hypothyroidism and goiter] (n = 4), and obesity (n = 3) [61, 63, 116]. Nevertheless, medical history was not reported for five PVT cases [3, 30, 62, 91, 98] and there were four PVT cases with previously established diagnoses of liver diseases [alcoholic cirrhosis (n = 2), nonalcoholic fatty liver disease (n = 1), and hepatitis C (n = 1)] [56, 57]. Radiological imaging shown PVT in almost all the patients who were included in this review and thought to have had developed PVTs post-COVID-19 vaccination [3–5, 30, 31, 49, 51–59, 61–67, 76, 91, 95, 96, 98, 109, 111], however, only a total of three cases presenting with PVT following COVID-19 vaccination were diagnosed based on liver histopathology [30, 54, 98, 116]. Patients who suffered PVT post-COVID-19 vaccination were more likely to have thrombocytopenia (n = 36) [3–5, 30, 31, 49, 51–55, 57, 58, 61–67, 76, 91, 95, 96, 98, 109, 111], high D-dimer (n = 34) [3–5, 30, 31, 49, 51–55, 57, 58, 61–67, 91, 95, 98, 109], positive antibodies directed against platelet factor 4 (n = 23) [3–5, 30, 49, 51–55, 57, 58, 61, 63, 65, 66, 76, 91, 111], high international normalized ratio (n = 10) [31, 53, 57, 63, 64, 67, 91, 98], high activated partial thromboplastin time (n = 8) [53, 54, 62, 67, 98, 109], low haemoglobin (n = 7) [61–63, 67, 76, 96, 98], and raised liver enzymes (n = 7) [51, 57, 58, 65, 67, 91, 96]. As expected, most prescribed pharmacotherapy agents in these PVT cases were the anticoagulants (n = 26, 51%), including unspecified type of heparins (n = 10), unspecified type of anticoagulants (n = 9), fondaparinux (n = 9), argatroban (n = 7), apixaban (n = 5), dalteparin (n = 3), rivaroxaban (n = 3), warfarin (n = 1), danaparoid (n = 1), or tinzaparin (n = 1) [3–5, 30, 31, 49, 51–53, 55–59, 61, 63–67, 76, 91, 96, 111, 116]. Many patients were also prescribed intravenous immunoglobulin (n = 19, 37.2%) [3–5, 30, 31, 49, 52, 53, 55, 57, 58, 61, 63, 65, 66, 76, 91, 111] and steroids (n = 11, 21.6%) [5, 49, 52, 57, 58, 61, 63, 64], however, pharmacotherapy was not reported in a high number of these PVT patients (n = 18, 35.3%) [54, 95, 98, 109]. Clinical outcomes of the PVT patients with mortality were documented in 25 (48.1%) [30, 31, 53, 54, 56, 61, 62, 64, 67, 95, 98, 109], while 23 (44.2%) of the PVT cases recovered [3–5, 49, 51–59, 61, 63, 65, 66, 76, 96, 111, 116] and few PVT patients were in a coma (n = 3, 5.9%) [64].

### Raised liver enzymes

Raised liver enzymes (RLEs) was the third most-common disease (twenty-six cases) reported following COVID-19 vaccination from our review (twenty-four new onset cases [9–11, 23, 25–28, 32, 33, 36, 38–40, 46, 70, 77, 79, 83, 94, 114, 121] and two relapsed cases [89, 93]) (see Table 1). Most common clinical presentations in those cases who presented with RLEs post-COVID-19 vaccination were fever (n = 11) [9, 10, 25, 28, 38, 70, 77, 79, 83, 114, 121], rash (n = 8) [25, 32, 38, 39, 79, 83, 89, 94], oedema (n = 8) [25, 32, 40, 79, 83, 89], weakness (n = 6) [26, 28, 46, 77, 79, 83, 89], fatigue (n = 5) [9, 25–27, 83], shortness of breath (n = 5) [26, 77, 78, 83, 89], vomiting (n = 5) [9, 11, 39, 77, 89], abdominal pain (n = 5) [39, 46, 83, 89, 121], headache (n = 5) [23, 33, 38, 83, 121], and myalgia (n = 4) [9, 25, 38, 77]. The median interquartile range (IQR) age of this group was 49 (32.7 to 68.2), with a similar gender rate in patients who presented with RLEs found after COVID-19 vaccination in all of the studies [female (n = 13) [10, 11, 27, 28, 32, 33, 36, 39, 40, 78, 83, 93, 94] and male (n = 13) [9, 23, 25, 26, 38, 46, 70, 77, 89, 114, 121]], and majority of the patients belonged to White (Caucasian) (n = 13, 50%) [9, 11, 23, 25–27, 32, 38, 46, 77, 83, 93, 94, 121] and Arab (n = 4, 15.4%) [39, 114] ethnicity. The median (IQR) time between the COVID-19 vaccination and time of presentation was 7 (4.5–11.5) days. Eleven, nine, and four of these twenty-five cases were reported following Pfizer-BioNTech (five after the first dose and six after the second dose) [9, 10, 25, 27, 28, 38–40, 79, 83, 93], Oxford Uni-AstraZeneca (eight after the first dose and one after the second dose) [23, 32, 33, 36, 46, 77, 114], and Moderna (four after the first dose) [26, 70, 89, 94] vaccination; respectively. Only two cases presented with RLEs were reported after Johnson & Johnson COVID-19 vaccination [11, 121]. Six of the patients who presented with RLEs had hypertension [11, 27, 33, 38, 83, 89] and nine patients had no medical history (n = 9, 34.1%) [9, 10, 26, 28, 32, 39, 40, 46, 121], however, few of those cases presented with a previous known history of hepatic diseases [chronic hepatitis B (n = 1) [83], alcohol-associated liver disease (n = 1) [70], chronic liver disease (n = 1) [36], portal hypertension (n = 1) [36], hepatitis C infection (n = 1) [89], and compensated alcoholic liver cirrhosis (n = 1) [25]]. Radiological imaging was unremarkable for a high number of the cases who presented with RLEs (n = 10, 40%) [11, 23, 25, 28, 38, 77, 79, 83, 93, 94] or not performed (n = 3, 12%) [10, 27, 36], nevertheless, few cases shown fatty liver and gallbladder polyps (n = 1) [70], liver cirrhosis (n = 1) [89], and abruptly collapsed hepatic veins (n = 1) [40]. Liver biopsy revealed histopathological findings consistent with leukocytoclastic vasculitis (n = 1) [36], drug reaction with eosinophilia (n = 1) [32], giant cell arteritis (n = 1) [23], plasmacytoid dendritic cells (n = 1) [10], and dermatomyositis (n = 1) [28]; however, histopathological examination was not performed in most of the cases (n = 18, %) [9, 11, 25–27, 33, 38, 39, 70, 77, 79, 83, 89, 93, 114, 121]. Patients who suffered RLEs post-COVID-19 vaccination were more likely to have high C-reactive protein (n = 14) [10, 11, 23, 25, 32, 33, 36, 38, 70, 89, 114, 121], thrombocytopenia (n = 13) [10, 11, 27, 33, 39, 83, 89, 93, 94, 114, 121], high lactate dehydrogenase (n = 11) [10, 11, 26, 27, 33, 36, 39, 46, 83, 89, 93], raised bilirubin (n = 10) [10, 11, 26, 27, 38, 39, 79, 83, 89, 114], low haemoglobin (n = 7) [11, 27, 33, 38, 39, 114], high creatinine (n = 6) [10, 11, 28, 33, 46, 77, 89], high reticulocyte count (n = 5) [26, 27, 39, 83, 93], high D-dimer (n = 5) [10, 33, 40, 46, 114], raised white blood cells (n = 4) [11, 27, 39, 77], high leukocytes (n = 4) [26, 33, 36, 79], and high ferritin (n = 4) [10, 38, 79, 89]. Most prescribed pharmacotherapy agents in patients with RLEs post-COVID-19 vaccination were steroids (n = 19) [9–11, 23, 25–28, 32, 33, 36, 38, 39, 46, 70, 78, 83, 93, 94], intravenous immunoglobulin (n = 8) [9, 10, 27, 28, 33, 40, 93, 94], and antibiotics (n = 7) [9, 46, 114, 121]. Clinical outcomes of the RLEs patients with mortality were documented in 2 (7.7%) [46, 114], while 22 (84.6%) of the RLEs cases recovered [9, 10, 23, 25–28, 32, 33, 36, 38–40, 70, 77, 79, 83, 93, 94, 114, 121] and final treatment outcome was not reported in two RLEs patients (n = 2, 7.7%) [11, 89].

### Acute liver injury

Acute liver injuries (ALIs) was the fourth most-common disease (twenty-one cases) reported following COVID-19 vaccination from our review [sixteen new onset cases [12, 13, 44, 99, 113, 122] and five relapsed cases [13]] (see Table 1). Most common clinical presentations in patients who presented with ALIs post-COVID-19 vaccination were abdominal tenderness (n = 3) [12, 113], jaundice (n = 2) [44, 113], yellow eyes (n = 2) [12, 44], weakness (n = 2) [12, 44], and vomiting (n = 2) [12, 113]. The median interquartile range (IQR) age of this group was 61 (41.5–68), with a female predominance in ALIs patients diagnosed after COVID-19 vaccination in most of the studies [n = 14, 66.7%] [12, 13, 99, 113, 122], and ethnicity was not reported for majority of the patients (n = 16, 80%) [13]. The median (IQR) time between the COVID-19 vaccination and time of presentation was 24 (7.5–31) days. Sixteen and four of these twenty cases were reported following Pfizer-BioNTech [12, 13, 99, 113, 122] and Moderna [13] vaccination; respectively. Only one case presented with liver injury was reported after Sinopharm COVID-19 vaccination [44]. Most of those cases presented with a previous known history of hepatic diseases [chronic liver disease (n = 6) [13], AIH (n = 4) [13], cirrhosis (n = 3) [13], hepatitis C virus (n = 1) [13], drug-induced liver injury (n = 1) [13], alcohol-associated liver disease (n = 1) [99], and liver transplant recipient (n = 1) [99]]. Radiological imaging was unremarkable for few cases who presented with ALIs (n = 4, 19%) [13, 99, 122], however, liver biopsy revealed histopathological findings consistent with AIH in one case [13] but biopsy examination was not made for many patients (n = 10, 47.6%) [13, 44, 99, 113, 122]. Patients who suffered ALIs post-COVID-19 vaccination were more likely to have raised liver enzymes (n = 20) [12, 13, 44, 99, 122], raised bilirubin (n = 15) [12, 13, 44, 99], high international normalized ratio (n = 8) [13, 113], positive antinuclear antibodies (n = 5) [13], and positive anti-smooth muscle antibodies (n = 4) [13]. Most prescribed pharmacotherapy agents in patients who suffered ALIs post-COVID-19 vaccination were steroids (n = 8) [13] and N-acetylcysteine (n = 3) [13, 113]. All patients who experienced ALIs after COVID-19 vaccination recovered (n = 21, 100%) [12, 13, 44, 99, 113, 122].

### Splanchnic vein thrombosis

Splanchnic vein thrombosis (SVT) was the fifth most-common disease (fourteen cases) reported following COVID-19 vaccination from our review (fourteen new onset cases [47, 48, 50, 60]) (see Table 1). Most common clinical presentations in patients who presented with SVT post-COVID-19 vaccination were abdominal tenderness (n = 2) [47, 50], fatigue (n = 2) [48, 60], nausea (n = 2) [47, 60], and headache (n = 2) [50, 60]. The median interquartile range (IQR) age of this group was 55 (48.2 to 61), with a female predominance in SVT patients diagnosed after COVID-19 vaccination in most of the studies (n = 12, 60%) [47, 48, 50, 60], and all patients belonged to the White (Caucasian) ethnicity (n = 20, 100%) [47, 48, 50, 60]. The median (IQR) time between the COVID-19 vaccination and time of presentation was 8.5 (6.7–13.2) days. Thirteen of these fourteen SVT cases were reported following Oxford Uni-AstraZeneca vaccination [47, 48, 60] and only one case presented with SVT was reported after Johnson & Johnson COVID-19 vaccination [50]. Unexpectedly, most of the SVT cases had no medical history (n = 11, 73.3%) [47, 48, 50, 60]. Radiological imaging for SVT patients shown cerebral venous thrombosis (n = 9) [60], disseminated intravascular coagulation (n = 5) [60] and pulmonary embolisms (n = 3) [60]. Patients who experienced SVT post-COVID-19 vaccination were more likely to have thrombocytopenia (n = 14) [47, 48, 50, 60], positive for antibodies directed against platelet factor 4 antibodies (n = 13) [48, 50, 60], high D-dimer (n = 10) [47, 48, 50, 60], high activated partial thromboplastin time (n = 6) [50, 60], high international normalized ratio (n = 5) [60], and low fibrinogen (n = 5) [50, 60]. Most prescribed pharmacotherapy agents in patients who suffered SVTs post-COVID-19 vaccination were the heparins (n = 7, 50%) [48, 50, 60], anticoagulants (n = 4, 28.6%) [47, 48, 50, 60], and intravenous immunoglobulin (n = 3, 21.4%) [47, 48, 50]. Clinical outcomes of the SVT patients with mortality were documented in 6 (42.8%) [60], while 8 (57.1%) of the SVT cases recovered [47, 48, 50, 60].

### Acute cellular rejection of the liver

Acute cellular rejection of the liver (ACRL) was the sixth most-common disease (eight cases) reported following COVID-19 vaccination from our review (six new onset and two relapsed cases [29, 34, 69, 82]) (see Table 1). The median interquartile range (IQR) age of this group was 59.5 (52.5–64.7), with a male predominance in ACRL patients diagnosed after COVID-19 vaccination in most of the studies [n = 5, 62.5%] [34, 69], and all patients belonged to the White (Caucasian) ethnicity (n = 8, 100%) [29, 34, 69, 82]. The median (IQR) time between the COVID-19 vaccination and time of presentation was 11 (7.5–17.2) days. Four of these eight ACRL cases were reported following Pfizer-BioNTech vaccination [29, 34, 69] and four of these eight ACRL cases were reported after Moderna COVID-19 vaccination [69, 82]. All of the ACRL cases had previous medical history related to the liver [non-alcoholic steatohepatitis-related cirrhosis (n = 3) [69], alcohol-related cirrhosis (n = 2) [69], history of acute cellular rejection (n = 2) [69], autoimmune cirrhosis (n = 1) [29], cryptogenic cirrhosis (n = 1) [34], cirrhosis (n = 1) [82], end-stage liver disease (n = 1) [29], hepatitis C virus (n = 1) [82], and hepatocellular carcinoma (n = 1) [82]]. Liver biopsy for the ACRL cases shown typical features consistent with acute liver rejection [mixed portal inflammation of predominantly mixed activated lymphocytes, bile duct injury, and endotheliitis] (n = 7, 87.5%) [29, 34, 69, 82]. Patients who experienced ACLR post-COVID-19 vaccination were more likely to have raised liver enzymes (n = 6) [29, 34, 69, 82], raised bilirubin (n = 5) [34, 69], and thrombocytopenia (n = 2) [29, 34]. Most prescribed pharmacotherapy agents in patients who suffered ACRL post-COVID-19 vaccination were the steroids (n = 12), IVIG (n = 2) [29, 34], immunosuppressants (n = 4) [tacrolimus(n = 2), everolimus (n = 1) and cyclosporine (n = 1)] [69], and mycophenolate mofetil (n = 2) [69, 82]. All patients who experienced ACRL after COVID-19 vaccination recovered (n = 8, 100%) [29, 34, 69, 82].

### Jaundice

Jaundice was the seventh most-common disease (eight cases) reported following COVID-19 vaccination from our review (six new onset cases [71–75, 81] and two relapsed cases [90, 125]) (see Table 1). The median interquartile range (IQR) age of this group was 55 [39 to 60], with a similar gender rate in patients who presented with jaundice found after COVID-19 vaccination in all of the studies [female (n = 4) [73, 75, 81, 90] and male (n = 4) [71, 72, 74, 125]], and most patients belonged to the White (Caucasian) ethnicity (n = 4, 50%) [73, 81, 90, 125] and Arab (n = 2, 28.6%) [71, 75] ethnicity. The median (IQR) time between the COVID-19 vaccination and time of presentation was 4 (2.2–9.2) days. Six and two of these eight jaundice cases were reported following Pfizer-BioNTech COVID-19 vaccination [72, 73, 75, 81, 90, 125] and Oxford Uni-AstraZeneca COVID-19 vaccination [71, 74]; respectively. Few of the jaundice cases had no medical history (n = 3, 37.5%) [72, 74, 75]. Patients who experienced jaundice post-COVID-19 vaccination were more likely to have raised bilirubin (n = 7) [72–75, 81, 90, 125], raised liver enzymes (n = 5) [72, 74, 81, 90, 125], thrombocytopenia (n = 4) [71, 72, 74], high reticulocyte count (n = 4) [71–73, 75], low Hb (n = 4) [71–73, 75], and high LDH (n = 3) [71, 74, 75]. Most prescribed pharmacotherapy agents in patients who suffered jaundice post-COVID-19 vaccination were the steroids (n = 4) [71–73, 81] and rituximab (n = 3) [71, 72, 75]. All patients who experienced jaundice after COVID-19 vaccination recovered (n = 7, 87.5%) [71–75, 81, 125] except one case who had a history of portal hypertension, hepatitis B and C, and hepatic cirrhosis and patient eventually expired [90].

### Acute hepatic failure

Acute hepatic failure (AHF) was reported in four cases following COVID-19 vaccination from our review [four new onset cases [35, 45, 78, 128]], with abdominal pain (n = 3) [45, 78, 128], nausea (n = 2) [35, 78], myalgia (n = 2) [45, 78], and fatigue (n = 2) [35, 45] as the common clinical presentations in these cases (see Table 1). The median patient age ranged from 24 to 53 years across studies. Two of the AHF cases were males and one patient was female [ethnicity: White (Caucasian) = 2 [35, 45] and Persian = 2 [78, 128]]. AHF occurred in patients within 1–10 days due to the use of Pfizer-BioNTech COVID-19 vaccination [35, 45] or Oxford Uni-AstraZeneca COVID-19 vaccination [78, 128]. Three of the AHF cases had no medical history (n = 3, 75%) [35, 45, 78]. Patients who experienced AHF post-COVID-19 vaccination were more likely to have raised liver enzymes (n = 4) [35, 45, 78, 128], raised bilirubin (n = 3) [45, 78, 128], and high INR (n = 3) [45, 78, 128]. The most prescribed pharmacotherapy agent in patients who suffered AHF post-COVID-19 vaccination was the steroids (n = 4, 100%) [35, 45, 78, 128], and one AHF patient received a new liver transplant [45]. Among these AHF patients, two patients survived [35, 45] and two patients deceased [78, 128].

### Hepatomegaly

Hepatomegaly was reported in three cases following COVID-19 vaccination from our review (three new onset cases [24, 88, 100]) (see Table 1). The median patient age ranged from 22 to 69 years across studies. All cases were females (n = 3, 100%) [ethnicity: White (Caucasian) = 2 [88, 100] and Indian = 1 [24]]. Patients developed hepatomegaly within 1–10 days after receiving Oxford Uni-AstraZeneca (n = 1) [100], Pfizer-BioNTech (n = 1) [88], and Covishield (n = 1) [24] COVID-19 vaccination. Two patients who developed hepatomegaly post COVID-19 vaccination had no medical history [88, 100], however, one patient had a history of infective jaundice [24]. Patients who experienced hepatomegaly post-COVID-19 vaccination were more likely to have thrombocytopenia (n = 2) [24, 100], high C-reactive protein (n = 2) [88, 100], high erythrocyte sedimentation rate (n = 2) [24, 88], and high lactate dehydrogenase (n = 2) [24, 100]. The most prescribed pharmacotherapy agent in patients who suffered hepatomegaly post-COVID-19 vaccination was the steroids (n = 3, 100%) [24, 88, 100]. All patients who experienced hepatomegaly after COVID-19 vaccination recovered (n = 3, 100%) [24, 88, 100].

### Hepatic porphyria

Hepatic porphyria was reported in a 34 year-old white female following the Oxford Uni-AstraZeneca vaccine, with development of abdominal pain, red urine, and hyponatremia, needing intensive care admission [one new onset case [92]] (see Table 1). Patient experienced syndrome of inappropriate antidiuretic hormone then acute hepatic porphyria was diagnosed, and the patient recovered completely after treatment with hemin [92].

## Discussion

A considerable range of liver diseases were observed following COVID-19 vaccination. As the dominant pathology reported in our review, AIH is defined as a chronic, inflammatory disease of the liver that is characterized by circulating autoantibodies and elevated serum globulin levels [129]. AIH occurs globally in all ethnicities and affects children and adults of all ages, with a female predominance [130]. A loss of tolerance against the patient’s own liver antigens is regarded as the main underlying pathogenetic mechanism, which is probably triggered by environmental agents such as pathogens and xenobiotics, in genetically susceptible individuals [130]. Although the mechanisms associated with COVID-19 vaccination and AIH are still unknown, molecular mimicry has emerged as the most likely process associated with this phenomenon [131]. Indeed, antibodies against the spike protein S1 of SARS-CoV-2 had a high affinity against some human tissue proteins [132]. As Pfizer-BioNTech, Oxford Uni-AstraZeneca, and Moderna vaccines code the same viral protein [133], they can trigger autoimmune diseases in predisposed patients. Diagnosis of AIH is based upon characteristic serologic and histologic findings and exclusion of other forms of chronic liver disease [134]. AIH can often be strongly suspected based upon clinical and laboratory features, and thus a liver biopsy is not always necessary in patients with typical findings on noninvasive testing [135]. Findings in liver biopsy correlate with reports of AIH following SARS-CoV-2 vaccination. Necroinflammatory hepatitis was observed in all cases of AIH following vaccination with Pfizer-BioNTech [6, 41, 43, 68, 84, 87, 99, 105, 106, 112], Moderna [7, 8, 80, 85, 97, 99, 102, 103, 107, 108], Oxford Uni-AstraZeneca [37, 86, 99, 101], Covishield [104] and Sinovac-CoronaVac [110] vaccine. AIH is a relatively rare; and AIH patients should receive anti-SARS-CoV-2 vaccination when the disease activity is controlled by immunosuppressive therapy [136]. Patients with new acute onset of AIH following anti-SARS-Cov-2 vaccine should be managed as suggested by current guidelines of American Association for the Study of Liver Diseases [137], British Society of Gastroenterology [138] and European Association for the Study of the Liver [139] that recommend the initial use of therapy with either glucocorticoid monotherapy or a combination of a glucocorticoid and azathioprine. The aim of treatment is induction of stable remission. Biochemical remission is defined as lowering of transaminase and immunoglobulin G levels to normal [130] and without treatment, the survival rate in patients with symptomatic AIH at five years is approximately 50 percent [140]. However, with treatment, the 10 year survival rate is approximately 90 percent [141]. Subsequent management will depend on how the patient responds to the initial treatment (remission, incomplete response, failed treatment, drug intolerance) and whether the patient relapses if treatment is withdrawn [137–139].

PVT is defined as the sudden onset of portal venous occlusion due to thrombus [142]. PVT can develop in the main body of the portal vein or its intrahepatic branches and may even extend to the splenic or superior mesenteric veins and occlusion may be complete or partial [142]. The pathogenesis of PVT associated with the use of COVID-19 vaccines against SARS-CoV-2 is suggested as the result of the viral proteins and free deoxyribonucleic acid in the vaccine binding to platelet factor 4 to generate a neoantigen that subsequently leads to the development of antibodies against platelet factor 4 which activate platelets and promote clotting [143]. It should be noted the risk of PVT after vaccination against SARS-CoV-2 do not appear to be higher than the background risks in the general population, a finding consistent with the rare and sporadic nature of this syndrome [54]. Anticoagulation therapy for patients with acute PVT due to COVID-19 vaccination is recommended [144]. Therapeutic anticoagulation is one of the primary treatments for PVT and is used unless there is a contraindication such as expanding intracerebral hemorrhage [144]. The choice of anticoagulant depends on the patient's clinical status and anticipated need to stop anticoagulation (based on risk of bleeding or need for an invasive procedure) [144]. Rapid anticoagulation can be achieved by starting PVT patient on low molecular weight heparin, with a switch to non-heparin anticoagulant agents, such as argatroban, danaparoid, fondaparinux, or direct oral anticoagulants (such as apixaban, edoxaban, or rivaroxaban) once the patient's condition has stabilized and no invasive procedures are planned [144]. Administration of intravenous immune globulin (IVIG) should not be delayed for PVT post-COVID-19 vaccination especially for individuals with thrombocytopenia [143]. Evidence supporting the use of IVIG comes from its use in other forms of autoimmune heparin induced thrombocytopenia which is the closest comparison to PVT, and IVIG would be expected to have direct antibody-mediated toxic effects [54]. Plasma exchange with plasma rather than albumin could also be effective in temporarily reducing levels of pathologic antibodies and providing some correction of the coagulopathy in terms of the hypofibrinogenemia [144]. Avoidance of platelet transfusions is critical, because such treatment would provide a substrate for further antibody-mediated platelet activation and coagulopathy [54].

RLEs post-COVID-19 vaccination led to nearly 74.5% of cases of liver injuries and about 3.8% cases of AHF. From all the spontaneous reports that we included in our review from patients who received Pfizer-BioNTech, Oxford Uni-AstraZeneca, Moderna, Johnson & Johnson, Sinovac-CoronaVac, Covishield, and Sinopharm vaccines worldwide between 1 December 2020 and 31 July 2022, there are reports of one hundred and six patients having abnormal liver function analysis [6–13, 23, 25–29, 32–46, 51, 57, 58, 60, 65, 67–70, 72, 74, 77–87, 89–91, 93, 94, 96, 97, 99, 101–108, 110, 112, 114] and out of these who had the RLEs there are seventy nine patients having COVID-19 vaccine-induced liver injuries [6–8, 12, 13, 29, 34, 35, 37, 41–45, 51, 57, 58, 60, 65, 67–69, 72, 74, 78, 80–82, 84–87, 90, 91, 96, 97, 99, 101–108, 110, 112] and ultimately four cases ending up with AHF [35, 45, 78, 99]. This systematic review shown the pooled incidence of cases with acute liver injuries diagnosed after COVID-19 vaccination was much higher in women [12, 13, 99, 113], which is consistent with a previously reported finding that shown female gender is more susceptible for drug-induced liver injury [145]. This may be related to the fact that these drugs often produce drug-induced liver injury with autoimmune features, and women are more susceptible to drug-induced AIH [146]. Liver injury, which is chronic in nature, increases in severity over time [147]. Cirrhosis, fatty liver, fibrosis and cancer are examples of chronic liver injuries. However, ALIs occur rapidly and may include COVID-19 vaccine-induced liver failure [147]. Serum levels of liver enzymes and bilirubin are commonly used for the noninvasive diagnosis of liver injury. But these diagnostic parameters are not specific in nature and cannot be used to identify a specific type of liver injury [148]. For instance, liver enzymes may increase in people due to no liver injury (e.g., alcohol, obesity or muscle damage) [149]. Furthermore, serum aminotransferase levels may rise too late for therapeutic intervention (e.g., acute toxicity of paracetamol) [150]. Therefore, serum RLEs and bilirubin may not delineate between different types of liver injury and do not always correlate well with the severity of the liver disease and prediction of clinical outcome; they are general rather than specific indicators. While it is important to recognize and treat RLEs after COVID-19 vaccination, it is equally important not to always label these infrequent cases with RLEs as serious, particularly when there are no objective findings. Most of the identified cases with RLEs post-COVID vaccination recovered and should not discourage vaccination against SARS-CoV-2.

Patients with chronic liver diseases (CLDs), particularly cirrhosis, hepatocellular malignancies, candidates for liver transplantation, and immunosuppressed individuals after liver transplantation appear to be at increased risk of COVID-19 infections, which in turn translates into increased mortality [151]. Therefore, vaccination against various diseases including COVID-19, administered as early as possible in patients with CLDs, is an important protective measure [152]. However, due to impaired immune responses in these patients, the immediate and long-term protective response through immunization may be incomplete [152]. Patients with advanced CLD have deficiencies in innate and humoral immunity [153, 154] and liver transplant recipients require immunosuppressant medications and have blunted antibody responses following SARS-CoV-2 vaccinations [155]. CLDs patients and liver transplant recipients were shown to develop substantially lower immunological response and undetectable or suboptimal poor antibody responses [155, 156] even after three doses of COVID-19 vaccine [157–159]. Currently, effective measures to improve immunogenicity to the COVID-19 vaccine in this population remain unknown and are urgently needed [155]. Although there may be big concerns that COVID-19 vaccines could lead to immunologically mediated rejection of the liver [29, 34, 69, 82], luckily, acceptance rate for COVID-19 vaccination among liver transplant recipients is extremely high [160, 161]. It is worth mentioning that several controlled trials and case series studies showed no increased risk of rejection with standard vaccination against SARS-CoV-2 compared with non-vaccinated controls [155, 162–166]. It is important to note that all cases of ACRL post-COVID-19 vaccination included in this review were easily treated without any serious complications and these findings should not be used to discourage vaccination for COVID-19 in patients with CLDs or liver transplant recipients [29, 34, 69, 82]. Vaccination against SARS-CoV-2 for patients with CLDs and hepatobiliary cancer, as well as for liver transplant recipients is recommended and should be prioritised in household members of patients with those liver pathologies, and in healthcare professionals caring for these patients [152].

SVT including portal, mesenteric, splenic vein thrombosis and the Budd-Chiari syndrome, is a manifestation of unusual site venous thromboembolism [167]. SVT presents with a lower incidence than deep vein thrombosis of the lower limbs and pulmonary embolism, with PVT and Budd-Chiari syndrome being respectively the most and the least common presentations of SVT [167]. SVT represents an extremely rare entity but which can be quite severe and worrisome for healthcare providers, and perhaps, not that “infrequent” [168]. Because almost all SVT and PVT cases reported post-COVID-19 vaccination occurred as a result of Oxford Uni-AstraZeneca vaccine use [3–5, 31, 47–49, 51, 52, 54–56, 58–62, 64, 65, 67, 76, 91, 95, 96, 98, 109, 111], while six PVT cases [30, 53, 57, 63, 66] and one SVT case [50] were reported after Johnson & Johnson COVID-19 vaccination, clinicians should be more suspicious to the scarce existence of PVT or SVT in patients with symptoms like severe abdominal pain, nausea or vomiting, fatigue, melena, and persistent high fevers within the setting of previous exposure to the Oxford Uni-AstraZeneca COVID-19 vaccine.

**Fig. 2:**
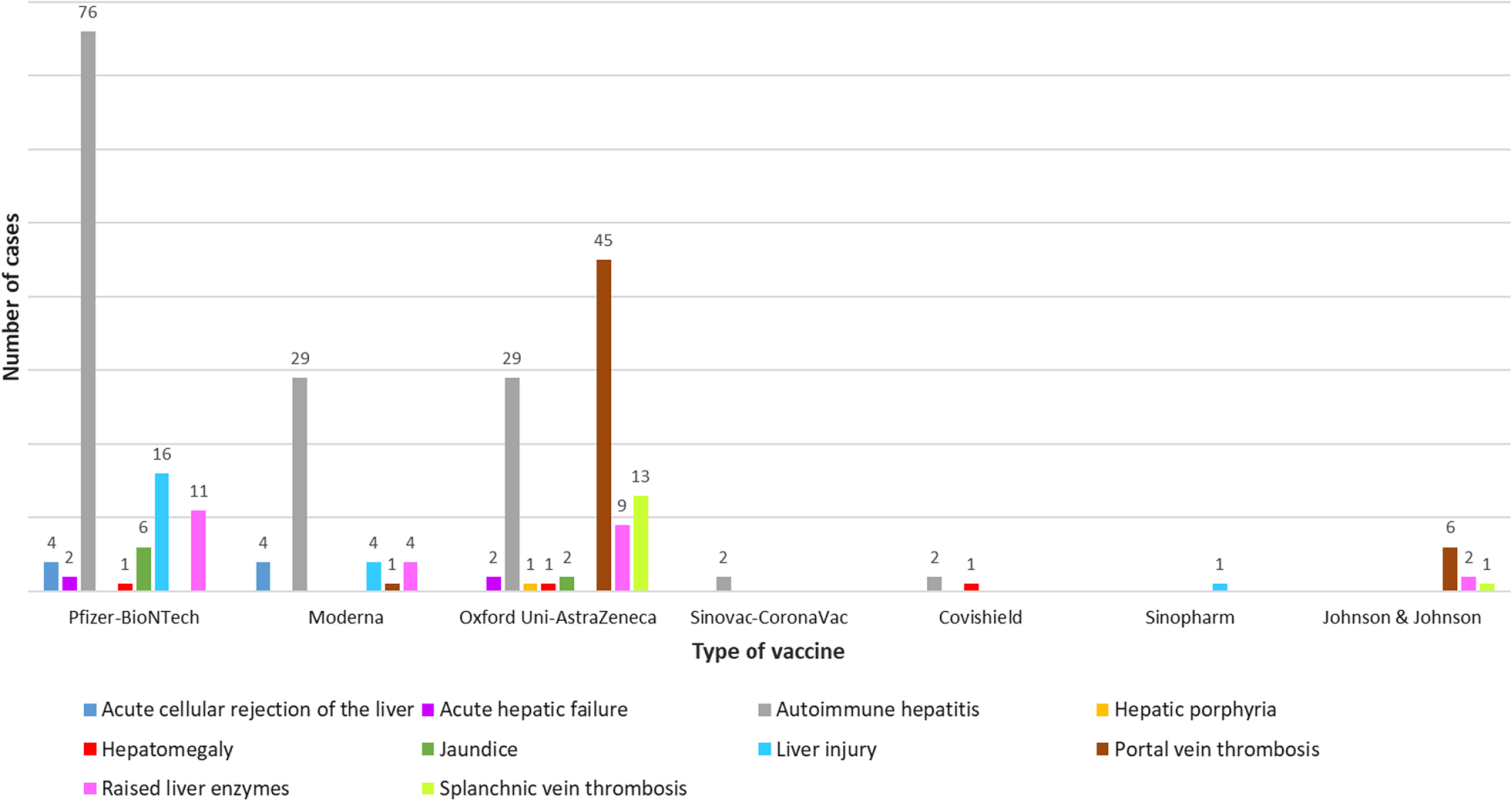
Summary of liver pathologies and the type of COVID-19 vaccines administered

From the one hundred seventy-three cases that were evaluated in our review, Oxford Uni-AstraZeneca (79 cases) [3–5, 23, 31–33, 36, 37, 46–49, 51, 52, 54–56, 58–62, 64, 65, 67, 71, 74, 76–78, 86, 91, 92, 95, 96, 98–101, 109, 111, 114], Pfizer-BioNTech (57 cases) [6, 9, 10, 12, 13, 25, 27–29, 34, 35, 38–41, 43, 45, 68, 69, 72, 73, 75, 79, 81, 83, 84, 87, 88, 90, 93, 99, 105, 106, 112, 113], and Moderna (24 cases) [7, 8, 13, 26, 69, 70, 80, 82, 85, 89, 94, 97, 99, 102, 103, 107, 108] appear to be the most frequent COVID-19 vaccines associated with post-vaccination liver disease development (see Fig. 2). The higher number of cases can be attributed to the immune response generated to those COVID-19 vaccines [131, 132, 143] or probably due to the fact that the vast majority of cases were reported from a select number of countries across North America, Europe, and Asia, where Oxford Uni-AstraZeneca, Pfizer-BioNTech and Moderna vaccines have been more accessible and commonly available in established vaccination programs [169, 170].

### Limitations

First, while most of the evidence discussed were based on few case series and many case reports, many of these are small and performed in single centers and not necessarily generalizable to the current COVID-19 vaccination settings. Second, all studies included in this review were retrospective in design which could have introduced potential reporting bias due to reliance on clinical case records. Third, the study population included adult patients and hence its results cannot be generalized to pediatric patients. Last, study was not registered in Prospero, an international prospective register of systematic reviews, as this might have added extra work and the merit was mostly limited to the avoidance of duplication.

## Conclusion

A range of liver diseases post-COIVD-19 vaccination may occur at extremely rare rate and is likely to be immune-mediated. Reported evidence of liver diseases post-COIVD-19 vaccination should not discourage vaccination. The number of reported cases is relatively very small in relation to the hundreds of millions of vaccinations that have occurred and the protective benefits offered by COVID-19 vaccination far outweigh the risks.

## Data Availability

Data are available upon request, please contact author for data requests.

## Abbreviations

ACRL: Acute cellular rejection of the liver
AHF: Acute hepatic failure
AIH: Autoimmune hepatitis
ALIs: Acute liver injuries
COVID-19: Coronavirus disease 2019
NOS: Newcastle–Ottawa scale
PRISMA: Preferred reporting items for systematic reviews and meta-analyses
SARS-CoV-2: Severe acute respiratory syndrome coronavirus 2
PVT: Portal vein thrombosis
RLEs: Raised liver enzymes
SVT: Splanchnic vein thrombosis
